# HyperTRIBE identifies hepatic IGF2BP2/IMP2 targets *in vivo* and links IMP2 to autophagy

**DOI:** 10.1093/narmme/ugag034

**Published:** 2026-06-24

**Authors:** Hoang Thu Trang Do, Simon Both, Tarek Kröhler, Marcello Pirritano, Elien Van Wonterghem, Elia Raab, Lisa Ahne, Sören Franzenburg, Emadeldin M Ibrahim, Martin Simon, Jeetayu Biswas, Volkhard Helms, Sonja M Kessler, Alexandra K Kiemer

**Affiliations:** Center for Bioinformatics, Saarland University, Saarbrücken 66123, Germany; Pharmaceutical Biology, Department of Pharmacy, Saarland University, Saarbrücken 66123, Germany; Pharmaceutical Biology, Department of Pharmacy, Saarland University, Saarbrücken 66123, Germany; Molecular Cell Biology and Microbiology, School for Mathematics and Natural Sciences, University of Wuppertal, Wuppertal 42119, Germany; VIB Center for Inflammation Research, Zwijnaarde 9052, Belgium; Institute of Pharmacy, Experimental Pharmacology for Natural Sciences, Martin Luther University Halle-Wittenberg, Halle 06120, Germany; Institute of Pharmacy, Experimental Pharmacology for Natural Sciences, Martin Luther University Halle-Wittenberg, Halle 06120, Germany; Institute of Clinical Molecular Biology, Kiel University, Kiel 24105, Germany; Center for Bioinformatics, Saarland University, Saarbrücken 66123, Germany; Molecular Cell Biology and Microbiology, School for Mathematics and Natural Sciences, University of Wuppertal, Wuppertal 42119, Germany; Department of Medicine, Department of Molecular Pharmacology, Memorial Sloan Kettering Cancer Center, New York, NY 10065, United States; Center for Bioinformatics, Saarland University, Saarbrücken 66123, Germany; PharmaScienceHub (PSH), Saarland University, Saarbrücken 66123, Germany; Pharmaceutical Biology, Department of Pharmacy, Saarland University, Saarbrücken 66123, Germany; Institute of Pharmacy, Experimental Pharmacology for Natural Sciences, Martin Luther University Halle-Wittenberg, Halle 06120, Germany; Halle Research Centre for Drug Therapy (HRCDT), Halle 06120, Germany; Pharmaceutical Biology, Department of Pharmacy, Saarland University, Saarbrücken 66123, Germany; PharmaScienceHub (PSH), Saarland University, Saarbrücken 66123, Germany; Centre for Gender-Specific Biology and Medicine (CGBM), Saarland University, Homburg 66421,Germany

## Abstract

Targets of RNA-binding proteins (RBPs) are often investigated by implementing variants of cross-linking and immunoprecipitation methodology, which can yield several disadvantages in target detection. The RBP and *N*6-methyladenosine (m6A) reader insulin-like growth factor 2 mRNA binding protein 2 (IGF2BP2/IMP2) exerts an essential pathophysiological role as a metabolic regulator and tumor promoter, impacting the stability, localization, and translation of its targets. Here, we employed HyperTRIBE as a method to identify RBP targets in native cells *in vivo* and identified targets of IMP2 in murine hepatocytes. IMP2-associated adenosine-to-inosine editing sites were identified by hydrodynamic transfection of mouse livers using an IMP2–ADAR (adenosine deaminase acting on RNA) construct. Functional enrichment and motif analysis results suggest IMP2-facilitated target stabilization and confirm presence of m6A-binding motifs. In addition, the overlap with data of a TRIBE experiment employing murine embryonic fibroblasts and with those of differential gene expression was investigated. Comparative transcriptomics between IMP2, wild-type, and control samples (mCherry–ADAR) revealed an enrichment of IMP2-bound mRNAs associated with autophagy, which could be validated by RNA immunoprecipitation in a human liver cancer cell line. A functional knockdown of IMP2 demonstrated an increased autophagic flux, providing further evidence for the involvement of IMP2 in autophagy.

## Graphical Abstract

**Graphical Abstract:**
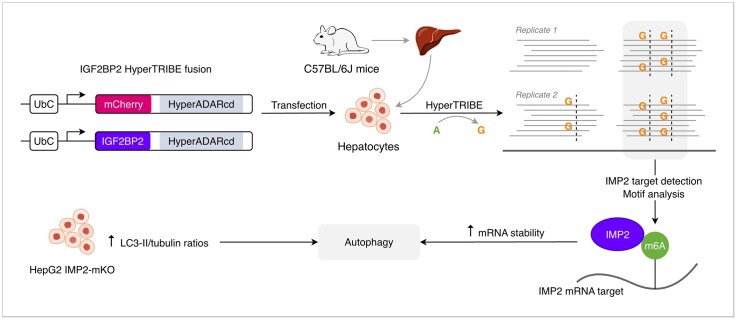

## Introduction

Insulin-like growth factor 2 mRNA binding protein 2 (IGF2BP2/IMP2) has originally been described as binding to the 5′-untranslated region (UTR) of the insulin-like growth factor 2 (IGF2) mRNA, but has meanwhile been suggested to bind to a broad range of RNAs [1–4] with IMP2 affecting the stability, localization, and translation of its target RNAs [5, 6]. IMP2 is known to exhibit an oncofetal expression pattern [7], whereby it controls the expression and translation of multiple oncogenes, thus promoting several hallmarks of cancer, including proliferation, migration, chemoresistance, and dysregulation of cellular metabolism, in gastrointestinal and other cancer entities [8–12]. As another deleterious effect, altered expression of IMP2 has been linked to the development of metabolic disorders [13, 14]. *In vivo* studies have demonstrated that transgenic IMP2 overexpression can induce a steatotic phenotype in mice [15] and can promote the progression toward steatohepatitis [16]. Furthermore, a murine liver-specific IMP2 overexpression model has also been shown to result in elevated hepatic iron deposition and increased abundance of free cholesterol in the liver [17]. Thus, IMP2 overexpression poses a putative method to model non-alcoholic fatty liver diseases [4, 18]. Recent studies have revealed that IMP2 binds to mRNAs containing *N*6-methyladenosine (m6A), the most prevalent modification in eukaryotic mRNAs [6]. Additionally, IMP2 was shown to stabilize USP13, which deubiquinates and thereby stabilizes autophagy-related protein 5 (ATG5) in an m6A-dependent manner [19].

Characteristic to all IMP proteins, there exist two RNA recognition motifs (RRMs), termed RRM1–2, at the N-terminus, and four K-homology (KH) domains at the C-terminus (KH1–4) [20]. Several RNA motifs are targeted by IMP family members [2, 20–22]. In heterogeneous nuclear ribonucleoproteins, it is known that RRMs and KH domains bind to short sequences of ~3–5 RNA bases [23, 24], yet few studies have elucidated specific binding motifs of RNA targets. For all members of the IMP family, the consensus binding motif CAUH was identified by a PAR-CLIP assay. The motif was detected in over 75% of the top 1000 targeted transcripts; a second CAU motif was detected in over 30% of transcripts in the dataset, predominantly within a distance of three to five nucleotides [3]. Concordantly, RNA bind-N-seq (RBNS) was conducted with recombinant full-length IMP1 and IMP2 to investigate preferred binding motifs, revealing CA-rich motifs to be enriched in a set of 3′ UTR-enriched targets in the eCLIP-defined binding sites [25]. However, Van Nostrand *et al*. compared five-mers in RBNS-bound sequences to corresponding enrichments in eCLIP peaks and procured different motifs utilizing these two techniques [26].

By implementing cross-linking and immunoprecipitation (CLIP) methodology to examine interactions between RNAs and RNA-binding proteins (RBPs), RBP–RNA complexes are subjected to immunopurification utilizing an RBP-specific antibody. As an alternative, RBP–RNA interactions can be directly monitored via TRIBE (targets of RBPs discovered by editing [27]), a protocol that is based on the expression of a fusion protein containing an RBP and the catalytic domain of the RNA-editing enzyme ADAR (adenosine deaminases acting on RNA). In the spatial vicinity of the expressed fusion protein, the deaminase domain of ADAR catalyzes the conversion of adenosine bases to inosine when bound to RNA. Such conversions can subsequently be detected by sequencing. The HyperTRIBE protocol [28] functions as a refined model of TRIBE, employing a catalytically hyperactive E488Q mutant of the ADAR protein to yield higher sensitivity with lower material concentrations; in contrast to CLIP, it cannot reveal exact RBP-binding positions on the mRNA [28]. HyperTRIBE reveals cell-specific RBP targets *in vivo*, [27] with suitable applications conducted *in vivo* in *Drosophila* [29] and *Plasmodium falciparum* [30] before, but not in mammals so far.

Here, IMP2-binding target mRNA molecules were determined in hepatocytes, as an important cell type for IMP2 action *in vivo*. We applied hydrodynamic gene delivery (HGD) in mice to specifically overexpress the ADAR-IGF2BP2 construct in hepatocytes. HGD is considered the most efficient nonviral technique for gene delivery into rodents because of its high efficiency, simplicity, safety, and reproducibility [31]. To this aim, as a completely novel application of this methodology in mice *in vivo*, HyperTRIBE editing sites in the livers of sham-treated mice were compared to those in livers of the HGD experimental groups: mice transfected with a plasmid encoding human IGF2BP2–ADAR or a control plasmid. The resulting findings on IMP2 targets were analyzed with respect to transcriptomic behavior. For comparison, HyperTRIBE editing sites were determined in mouse embryonic fibroblasts (MEFs), as well.

## Materials and methods

### Plasmid generation

TdMCP-stdGFP (Addgene #98916) was modified to generate the plasmids used in this study, as previously described by Biwas *et al*. [32]. Briefly, EGFP was replaced with dADAR E488Q with synonymized serine 458 codon. A p2A sequence was added to the 5′ end of dADAR via polymerase chain reaction (PCR). Ultimately, MCP was replaced with mCherry or human IMP2. Plasmid maps for the control plasmid, mCherry–ADAR (Addgene #154786), and the experimental plasmid, IMP2–ADAR (Addgene #247301), are shown in Supplementary Fig. S1. Plasmid isolation from *Escherichia coli* was performed according to the manufacturer’s instructions using the EndoFree Plasmid Giga Kit (#27106, #12143, #12391 Qiagen, Hilden, Germany). The concentration of the isolated plasmid DNA was determined by measuring the absorbance at *λ* = 260 nm, while quality was ensured by computing the purity ratio absorbance at *λ* = 260 nm/absorbance at *λ* = 280 nm with a NanoDrop Lite Spectrophotometer (#ND-LITE, Thermo Fisher Scientific).

### *In vivo* hepatocyte transfection

Animal handling was conducted in accordance with the guidelines of the local animal welfare committee (permission number: 04/2020). Mice were housed in groups of five under controlled conditions, with exposure to a daily 12 h light/12 h dark cycle at a temperature of 22°C ± 2°C and a relative humidity of 55% ± 10%, with access to food and water *ad libitum*.

Initiating *in vivo* transfection, 12- to 14-week old C57BL/6J mice were immobilized in a restrainer and rapidly injected (within 5 s) with 2.5 ml of 12.5 µg/ml sterile, endotoxin-free solution of plasmid DNA (Supplementary Fig. S1) in 1× phosphate buffered saline (PBS; #TMS-012-A, Merck) into the lateral tail vein. As a sham treatment, wild-type control animals were injected with 1× PBS. Prior to injection, to improve blood circulation, the mice were placed under an infrared lamp. In accordance with the local animal welfare officer the originally planned injection volumes of 2.6–3.2 ml, corresponding to 10% of animal body weight, were reduced to 2.5 ml due to a poor health outcome of the first injected mouse after administration of 3 ml of sterile, endotoxin-free 1× PBS. This mouse showed no spontaneous mouse behavior until euthanasia 1 h post-injection. Thus, an injection volume of 2.5 ml was the resulting compromise to ensure mouse health and to create a sharp increase in venous pressure for the uptake of foreign plasmid DNA. This approach was subsequently implemented on a total of nine animals, consisting of three experimental groups, with three mice in each group (*n* = 3): wild type, mCherry, and IMP2. At 1, 6, and 24 h post-injection, the health status of the mice was inspected. Mice were euthanized 24 h after injection.

Directly after liver removal, one-sixth of the left lateral lobe was embedded in Tissue-Tek^®^ O.C.T. Compound (#4583, Sakura Finetek Europe B.V., The Netherlands) and slowly frozen air bubble-free in liquid nitrogen. From these samples cryo-sections were prepared and analyzed via fluorescence microscopy. One-third of each liver lobe, consisting of the left and right lateral lobe, left and right medial lobe, and caudate lobe, was transferred into neutral-buffered paraformaldehyde [4% (w/v) in 10 mM PBS (1×)] for downstream tertiary butyl alcohol dehydration and paraffin embedding, following a protocol according to Zhanmu *et al*. [33]. Another third of each lobe was frozen in liquid nitrogen for subsequent RNA isolation and quantitative polymerase chain reaction (qPCR) analysis. Remaining liver slices were stored briefly in cold RPMI-1640 medium prior to hepatocyte isolation, followed by digestion with the use of a Liver Dissociation Kit mouse (#130-105-807, Miltenyi Biotec B.V. & Co. KG, Bergisch Gladbach, Germany). After digestion, the liver cells were resuspended in 1× PBS and subsequently analyzed by fluorescence-activated cell sorting (FACS), using the GFP and PE-CF594 channels.

### Histology

For histological analysis, paraffin-embedded liver tissue specimens were sliced into 5-µm sections and subjected to several immunohistochemical analyses, carrying out antibody staining using EnVision^®^+ Dual Link System-HRP (DAB+) (#K406511-2, Agilent Technologies, CA, USA) for GFP (recombinant anti-GFP antibody, #ab183734, [EPR14104], Abcam, Cambridge, United Kingdom), mCherry (Anti-mCherry antibody, #ab125096, [1C5], Abcam), and IMP2 (anti-p62C antibody from Lu *et al*. [34]), as described in a previous study [35]. The counts of positive hepatocytes were histologically scored in a blinded manner.

### RNA isolation, reverse transcription, and qPCR

RNA isolation from murine liver tissue (*n* = 3) was performed by homogenizing snap-frozen liver tissue in QIAzol^™^ lysis reagent (#79306, Qiagen) using a high-performance disperser (T 25 digital ULTRA-TURRAX^®^, IKA^®^, Staufen, Germany) prior to total RNA extraction with the Direct-zol™ RNA Miniprep Plus Kit (#R2071, Zymo Research Europe, Freiburg, Germany), according to the manufacturer’s protocol. Contamination of the RNA by residual genomic DNA was removed by DNase I treatment, using the DNA-free™ DNA Removal Kit (#AM1906, Thermo Fisher Scientific). RNA concentration was determined by measuring absorption at *λ* = 280 nm using a NanoDrop™ Lite spectrophotometer (Thermo Fisher Scientific), with RNA quality computed by the absorption ratio of *A*_260_/*A*_280_ . Samples with ratios above 1.9 were included in downstream analyses. RNA content of all samples was normalized prior to reverse transcription via the High Capacity cDNA Reverse Transcription Kit (#4368813, Thermo Fisher Scientific), following the manufacturer’s instructions, with the RNase inhibitor RNaseOUT™ (#10777019, Thermo Fisher Scientific) employed in all reactions.

qPCR analysis of cDNA was conducted at a 20-µl scale using the ready-to-use HOT FIREPol^®^ EvaGreen^®^ qPCR Mix Plus (#08-25-00020, Solis BioDyne), mixed with respective primers for the target transcript. All primer sequences and the annealing conditions are listed in Supplementary Table S1. Samples were analyzed in triplicate. The reaction was performed in a CFX96 touch™ Real-Time PCR detection system (BioRad Laboratories, Hercules, CA, USA), with the resulting data recorded with the CFX Manager™ software (version 3.1). Data were normalized relative to *Rn18s* as housekeeping gene. Standard plasmids were generated by ligation of gel-purified PCR amplicons of the gene transcripts of interest into the pGEM-T^®^ Easy vector (#A137A, Promega, Madison, WI, USA), according to the manufacturer’s instructions.

### RNA isolation, library preparation, and sequencing

RNA-seq was performed on samples derived from the right lateral lobe (*n* = 3). RNA was isolated from livers by adding 1.5 ml cold Tri-Reagent (Sigma) to 10 mg frozen liver tissue. Mechanical disruption of tissue was achieved by thawing tissue in a Qiagen Tissue Lyser, using tungsten carbide beads. Following protocol, RNA was isolated using phase separation. After DNAse digestion and additional purification with acid phenol, integrity was validated using the Agilent Bioanalyzer RNA Pico Chips.

For Illumina library preparation, the Qiagen FastSelect rRNA blockers were combined with the Diagenode D-plex total RNA library kit to create total RNA libraries. Using 50 ng total RNA, an RNA fragmentation time of 3.5 min was applied with subsequent RNA 3´-adenylation. Qiagen FastSelect rRNA blockers (1 µl of a 10-fold dilution) were added to the reverse transcription, which was carried out according to the manufacturer’s protocol. PCR amplification included 13 cycles. Libraries were size-selected using AmpureXP beads (Beckman) and measured for length distribution using the Bioanalyzer DNA High Sensitivity Chips. Sequencing was carried out at the Competence Centre for Genomic Analysis (CCGA) in Kiel, Germany on an Illumina Nova Seq 6000, using S4 flow cells in 2x100 PE mode. Sequencing data are available in the ArrayExpress database (http://www.ebi.ac.uk/arrayexpress) under accession number E-MTAB-14301.

### Transcriptomic data processing

The main steps in the bioinformatic analysis used for the identification of IMP2 binding targets from transcriptomic data include two parts, namely mRNA data preprocessing and IMP2 target genes detection and analysis (Supplementary Fig. S2). In the preprocessing of mRNA reads, we first extracted the unique molecular identifiers (UMIs) from the first 16 base pairs of the raw FASTQ-formatted forward reads with the *umitools extract* command [36]. Using *Cutadapt* [37], sequencing adapters were trimmed from paired reads with marked UMIs, simultaneously removing poly-A tails according to the protocol (Diagenode). Reads shorter than 20 base pairs were ultimately discarded from the final library. The trimmed reads were aligned against the *Mus musculus* reference genome version GRCm38 (mm10) in Refseq [38], using the *STAR* aligner [39]. The processed reads were subsequently sorted with the *samtools sort* command [40]. Finally, the aligned reads were deduplicated using the umitools dedup command and quantified with the transcript quantifier *Salmon* [41].

The homogeneity of WT, mCherry, and IMP2 samples were examined by computing either Pearson’s or Spearman correlation coefficients on the dataset, respectively, followed by applying principal component analysis to the log-transformed gene expression data for the replicates. Pairwise differential gene expression analysis was performed with *DESeq2* [42], whereby the gene expression data in WT, mCherry, and IMP2 samples were normalized, transformed, and compared combinatorically—or between each sample pair.

### Identification of IMP2 binding targets by HyperTRIBE

IMP2-targeted sites were identified in the processed transcripts using the software tool HyperTRIBE [43], available at https://github.com/rosbashlab/HyperTRIBE/. Initially, HyperTRIBE generates lists of editing sites, which are identified by comparing the controls against an experimental group. Then, the editing sites are filtered on the basis of coverage exceeding a certain threshold for the average editing percentage at 0%, 1%, or 5%. The resulting editing sites are reported with genome coordinates in *bedgraph* output files. As editing percentage indicates the fraction of reads with adenosines deaminated into inosines among total reads at a specific site [43], the high-stringency threshold of 5% requires a site to be edited in at least 5% of all transcripts. Furthermore, all sites retained after filtering with thresholds of 1% and 5% were required to have a coverage of at least 20 reads. For each comparison, transcripts with editing sites that remain after filtering with different editing thresholds are reported in Supplementary File S1. The total number of transcripts, total number of editing sites, average number of editing sites per transcript for each comparison, and editing thresholds are also summarized in Supplementary Table S2.

The edited sites identified by HyperTRIBE were sequentially refined in three steps. First, various replicate collapsing schemes were applied to combine A2G sites that distinguish WT, mCherry, and IMP2 samples, which were identified in multiple pairwise replicate comparisons. Background correction of identified IMP2 targets was sequentially performed to exclude results relevant only to control samples. Lastly, a final replicate collapsing scheme was selected, featuring high sensitivity and robustness based on the concordance between HyperTRIBE analysis results for mouse liver and MEFs, as well as between IMP2-target genes and deregulated genes. The selection strategy for the appropriate replicate collapsing scheme is shown and discussed in detail in the “Replicate collapsing scheme selection” section of Supplementary Information S1.

#### Replicate-collapsing schemes

Each replicate in the wild-type, mCherry, and IMP2 groups was compared against all replicates from other groups. Based on the documentation of HyperTRIBE [43], several schemes were incorporated to select common IMP2 targets that were identified consistently in comparisons of replicates belonging to two different groups. These replicate-collapsing schemes combine nearby editing sites across samples in several ways. This matches the experimental condition in which ADARcd only marks the vicinity of IMP2-binding sites with A2G sites, as opposed to competing with IMP2 for the exact binding sites [26]. Along with the resulting editing sites from HyperTRIBE analysis, we examined editing regions in which multiple sites were detected. Three editing regions were considered: (i) gene spans of size 10 bp, (ii) coding sequences (CDS) or UTRs, and (iii) full transcripts. In case (i), the 5-bp stretch up- or downstream of a detected editing site was identified using the *slop* command from *bedtools* [44]. The resulting editing sites or regions were either included or excluded using one of two techniques, shown in Supplementary Fig. S3, using the INTERSECT scheme, in which an editing site is required to be present in all three replicates of an experiment group, or using the UNION scheme, in which a site must be found in at least two of three replicates. The overlapping sites were extracted using the *intersect* command in *bedtools*, while duplicated sites and regions were merged using the *groupby* command. As HyperTRIBE compares each replicate from the first sample type to each replicate from the second sample, the results for the replicates of the second group were collapsed, as well as subsequently for the first group, to obtain the coordinates of editing sites and regions found between two sample types. If a region is retained, all editing sites detected in the genome span will be included in the final set of outcomes, as HyperTRIBE can only summarize the results based on sites, as opposed to genomic regions.

#### Background correction

After applying replicate collapsing schemes, the A2G sites or regions found in all replicate comparisons were filtered and merged for each pair of samples among WT, mCherry, and IMP2. As the focus remains on altered binding sites in IMP2 samples, the set of IMP2 targets between IMP2 and WT or mCherry were considered, excluding those only related to control groups. This resulting group is referred to as “background corrected IMP2-specific genes,” with the label “WT/mCherry versus IMP2 - WT versus mCherry.” More information on these genes in further analyses is provided in the “Background activity of ADAR” section of Supplementary Information S2.

The set of differentially expressed genes (DEGs) identified from the comparisons between IMP2 and either WT or mCherry in the previous section was corrected in a similar manner. Here, the background genes are deregulated genes detected by comparing two control samples: WT and mCherry. These genes were removed from IMP2-related DEGs and the results were termed as “background corrected DEGs.”

#### Concordance between IMP2 targets

- Overlap between HyperTRIBE results in mouse liver and MEFs. HyperTRIBE was employed to identify editing sites between a control and an experimental group, in the case of IMP2 against wild type or mCherry, or between the two controls, such as with mCherry against wild type. The *perl* script *summarize_results.pl* provided by HyperTRIBE generates a list of genes that possess edited adenosine sites, with the number of editing sites and the average editing percentage. To examine and compare the robustness of the replicate collapsing schemes, the gene set derived from a specific replicate-collapsing scheme was compared to the HyperTRIBE results for MEF samples; see the “Cell culture, transfections, and FACS sorting for MEF HyperTRIBE” section. The Jaccard Index was computed as a measure to examine similarity between sets of the same average editing percentage threshold, as well as the number of overlapping genes and its percentage in the newly generated gene sets.
- Overlap between HyperTRIBE results and DESeq2 DEGs. Next, the similarity was measured between the set of HyperTRIBE genes containing at least one A2G site and the set of DEGs, according to DESeq2 by the Jaccard index.
- We finally performed validation experiments (RNA immunoprecipitation) using deregulated IMP2 target genes identified from overlapping HyperTRIBE and DESeq2 results in each pairwise comparison across WT, mCherry, and IMP2, as well as after background correction.

### Analysis of HyperTRIBE results

#### Gene ontology enrichment analysis

Gene ontology enrichment analysis for biological processes was performed on DEGs using the R package *ClusterProfiler* [45]. Similar to DEGs, HyperTRIBE hits were subjected to gene ontology enrichment analysis. The analysis was performed for the sets of IMP2-target genes identified when IMP2 was compared to WT or mCherry, background genes from WT and mCherry comparisons, and background-corrected IMP2-specific genes.

#### Profiling of identified editing sites

Preferential editing regions were identified for detected IMP2 targets by annotating ascertained sites to the CDS, 3′, and 5′ UTR regions. As WT and mCherry were both used in our experiment as control samples for HyperTRIBE detection of IMP2-binding sites, the concordance of the results was analyzed when either one of the controls was compared to the IMP2 sample or when the controls were compared to each other, respectively. To this aim, the resulting genes were plotted using the number of detected A2G sites obtained via the various aforementioned pairwise comparisons. These plots reveal whether control samples display similar differences with respect to IMP2 and DEGs, as well as whether the number of editing sites detected for a gene is over- or underestimated in a comparison, respectively. Using these plots, genes marked with high editing frequency in the comparisons between both control groups against IMP2 were detected.

#### Effects of IMP2 binding on targeted transcripts

To inspect the stabilizing effect of IMP2 on targets reported in a previous study [12], it was investigated whether the expression levels of IMP2 target genes (IMP2+) would be more stable compared to genes without A2G editing sites (IMP2−). Thus, the cumulative distribution of Log Fold Changes (LFCs) of these IMP2+ and IMP2− genes was plotted, and Kolmogorov–Smirnow tests were performed to determine whether LFC distributions are significantly different. The LFCs were calculated using WT as the control in any comparison against WT, and using mCherry as the control in *mCherry* versus *IMP2* comparison. Additionally, we used Spearman correlation to measure the association between IMP2 and other genes, including either targets and non-target transcripts, with the assumption that RBP–mRNA binding dynamics exhibit non-linear relationships.

#### Motif enrichment analysis

IMP2 belongs to the set of m6A readers and exerts its stabilizing activity on transcripts by promoting m6A modification [46, 47]. This serves as motivation to investigate IMP2 editing sites and their proximity to enriched binding motifs. Based on a previously reported observation that 72% of HyperTRIBE editing sites overlapped with CLIP sites found in the 500-bp range [43], sequences were analyzed in a [−500 bp, +500 bp] region around each A2G site. Motif enrichment analysis for each HyperTRIBE comparison (WT versus IMP2, mCherry versus IMP2, and WT versus mCherry) was performed using HOMER, using a motif search window of size 200 bp [48]. Differential motif discovery was performed for the comparisons of WT versus IMP2 and mCherry versus IMP2 using HOMER. The identified motifs were considered significantly enriched if they had an adjusted *P*-value <1e−10, according to HOMER.

### Cell culture, transfections, and FACS sorting for MEF HyperTRIBE

Primary MEFs were isolated from E14 IMP2 KO embryos [49] and subsequently immortalized by transient transfection, with a plasmid expressing SV40 large T antigen (Addgene #21826). Single cell clones were then isolated by limiting dilution. MEFs were continuously cultured in DMEM (4.5 g/l glucose, Corning) supplemented with pen/strep (Gibco) and 10% fetal bovine serum (Atlanta Biologics). Transient transfection of TRIBE plasmids (Addgene #154786 and #247301; for more details see the “Plasmid generation” section) was carried out in two biological replicates (*n* = 2) and performed with JetPrime (Polyplus) in accordance with manufacturer instructions 12 h before sorting. Immediately before sorting, cells were trypsinized, pelleted for 5 min at 500 × *g* and pellets were resuspended in sorting buffer (DPBS, without calcium and magnesium, supplemented with 1% BSA and DAPI) before being passed through a single cell strainer. FACS was performed by selecting for GFP-positive, DAPI-negative single cells on a BD Aria II flow cytometer. A minimum of 10 000 GFP-positive cells were directly sorted into 800 μl Trizol (Invitrogen), with RNA isolation being subsequently performed as per manufacturer’s instructions.

#### RNA isolation and library prep from MEFs

GFP+/DAPI− MEFs were sorted via FACS directly into Trizol and RNA was extracted, quantified with qubit HS RNA assay; resulting integrity was visualized with an RNA pico chip (Agilent Bioanalyzer). Samples with no apparent degradation were used to prepare stranded libraries (NEB Ultra II Stranded RNA-seq library prep kit with poly A RNA isolation module) with 150 ng RNA input per cell, as quantified by qubit RNA HS assay. Final libraries were amplified with one to two rounds of PCR less than the manufacturer’s recommendation and validated on an Agilent bioanalyzer for appropriate size distribution. Library concentrations were then quantified by a qubit dsDNA HS assay, qPCR, and an Agilent bioanalyzer (performed by Novogene).

#### Sequencing and analysis of MEF HyperTRIBE data

Samples were combined and multiplexed across multiple lanes for HiSeq 4000 runs (performed by Novogene). Paired-end sequencing was performed with 150-bp reads at a depth of at least 27.5 Gb per sample, corresponding to at least 80 million reads per sample. The data were analyzed following the publicly available computational pipeline developed for HyperTRIBE [43]. Reads from sequencing libraries were trimmed and aligned to the UCSC mm10 reference genome. Subsequently, the nucleotide frequency at each position in the transcriptome is recorded from aligned reads. For each nucleotide in the transcriptome, the HyperTRIBE RNA nucleotide frequency was compared to the wild-type mRNA library (wtRNA) nucleotide frequency to identify RNA editing sites. For a legitimate site, the frequency of A is greater than 80% and the frequency of G is 0.5% in the wtRNA, while the frequency of G is greater than 0 in the HyperTRIBE RNA, using the reverse complement if the annotated gene is in the reverse strand. Modifying the nucleotide frequency of G from 0% to 0.5% in wtRNA allows for very low sequence heterogeneity in wtRNA and does not disrupt the identification of legitimate editing sites in deeply sequenced libraries. An editing threshold of at least 1% in control samples or 5% in experimental samples was instated in each replicate. Using above thresholds, the IMP2 HyperTRIBE editing sites were required to be present in both IMP2 replicates at >5% editing; all sites that overlap with editing sites from mCherry–ADAR with at least 1% editing are removed. The transcripts containing editing sites are listed in Supplementary File S1.

### Cell lines

The hepatocellular carcinoma cell lines HepG2 and Huh7 were used for cell-based experiments, also employing a monoallelic IMP2 knockout (HepG2 *mKO*) mutant with reduced IMP2 expression [10]. Cells were maintained in Dulbecco’s modified Eagle’s medium (DMEM), supplemented with 10% fetal calf serum (FCS), 1 mM glutamine, 100 U/ml penicillin, and 100 µg/ml streptomycin, unless stated otherwise. Cells were incubated at 37°C, with 5% CO_2_.

### Western blot

Fragments of left lateral liver lobes (30–50 mg) were homogenized in 200 µl of RIPA lysis buffer using a micro pestle, supplemented with a protease inhibitor (#04693124001, Roche). Liver tissue from embryonic mice at day 12.5, sourced from a previous publication [7], was used as a positive control for endogenous IMP2. The lysates were centrifuged at 20 000 x *g* for 25 min, and the resulting clear supernatant was diluted 1:10 in RIPA buffer. The final samples were mixed with loading buffer (#K929.1, Roth) and heated at 95°C for 10 min. A total of 10 µg of protein was loaded into an SDS–PAGE (sodium dodecyl sulfate–polyacrylamide gel electrophoresis) analysis. HepG2 *wildtype* and *monoallelic IMP2* knockout cells were seeded in six-well plates at a density of 700 000 cells/well, with cell culture media supplemented with 1% FCS. At 24 h after seeding, cells were treated with 10 nM bafilomycin A1 from *Streptomyces griseus* (#B1793, Sigma). At 0–4 h post-seeding, cells were washed with PBS and lysed in 200 µl lysis buffer (50 mM Tris, 1% SDS, 10% glycerol, 5% β-mercaptoethanol), supplemented with protease inhibitor (#04693124001, Roche). After performing sonication on the lysates, 25 µl of the lysate was used to conduct an SDS–PAGE analysis. After performing SDS–PAGE, proteins were blotted onto a 200 nm PVDF membrane (#1704156, BioRad) using the “mixed molecular weight” protocol of the Trans-Blot Turbo Transfer System (BioRad). The membrane was incubated in blocking solution (#MB-070, Rockland) for 1 h before being incubated with primary antibodies for at least 90 min. Antibodies used were specific for LC-3 (#2775, Cell Signaling), α-tubulin (SAB3501072, Sigma–Aldrich), and p62/IMP2 [34]. IRDye680-conjugated anti-rabbit IgG (#92668071, LI-COR Bioscience) and IRDye800- conjugated anti-mouse IgG (#926-32210, LI-COR Biosciences) were used as secondary antibodies. Signal intensities were determined using the Odyssey near-infrared imaging system (Li-COR Bioscience) and quantified using the Image Studio software (Li-COR Bioscience).

### RNA immunoprecipitation

RNA immunoprecipitation (RIP) of IMP2-associated RNAs from Huh7 cells was performed using Magne™ Protein A Beads (#G8782, Promega). Beads were pre-washed with PBST and incubated with either IMP2 antibody (5 µg, #RN008P, MLB) or rabbit IgG control (5 µg, #I5006, Sigma–Aldrich) for 90 min in 150 mM NaCl. Huh7 cells (5 × 10⁶) were harvested by centrifugation (500 x *g*, 5 min, 4°C), washed with ice-cold PBS, and lysed in 0.5 ml RIPA buffer (50 mM Tris–HCl, pH 7.5, 1% Nonidet *P*-40, 0.5% sodium deoxycholate, 0.05% SDS, 1 mM ethylenediaminetetraacetic acid , 150 mM NaCl in DEPC-treated water). After 10 min on ice, lysates were centrifuged (16 000 × *g*, 10 min, 4°C) and supernatants were incubated with antibody-loaded beads for 1 h at room temperature under rotation. Beads were washed three times with PBST and transferred to new reaction tubes prior to the final wash. RNA was isolated using the High Pure RNA Isolation Kit (#11 828665001, Roche). Briefly, 200 µl of PBS was added to the beads, followed by vortexing, centrifugation, and the addition of 400 µl of lysis buffer. The suspension was incubated overnight at −80°C, beads were magnetized for 15 min, and the supernatant was loaded onto the column. RNA purification was then completed according to the manufacturer’s protocol. RIP efficiency was assessed via qPCR, using GAPDH as a negative control. All buffers contained RNaseOUT and 7× Complete protease inhibitor. The sequences of the primers used are listed in Supplementary Table S1.

## Results

### Hepatocyte *in vivo* transfection

Three wild-type male C57BL/6J mice, each, were hydrodynamically injected to be transfected with either a control plasmid (mCherry–ADAR) or experimental (IMP2–ADAR) plasmid DNA, and livers were removed 24 h post-injection, with transfection efficiency determined afterward; plasmid sequence can be found in Supplementary Fig. S1.

**Figure 1.**
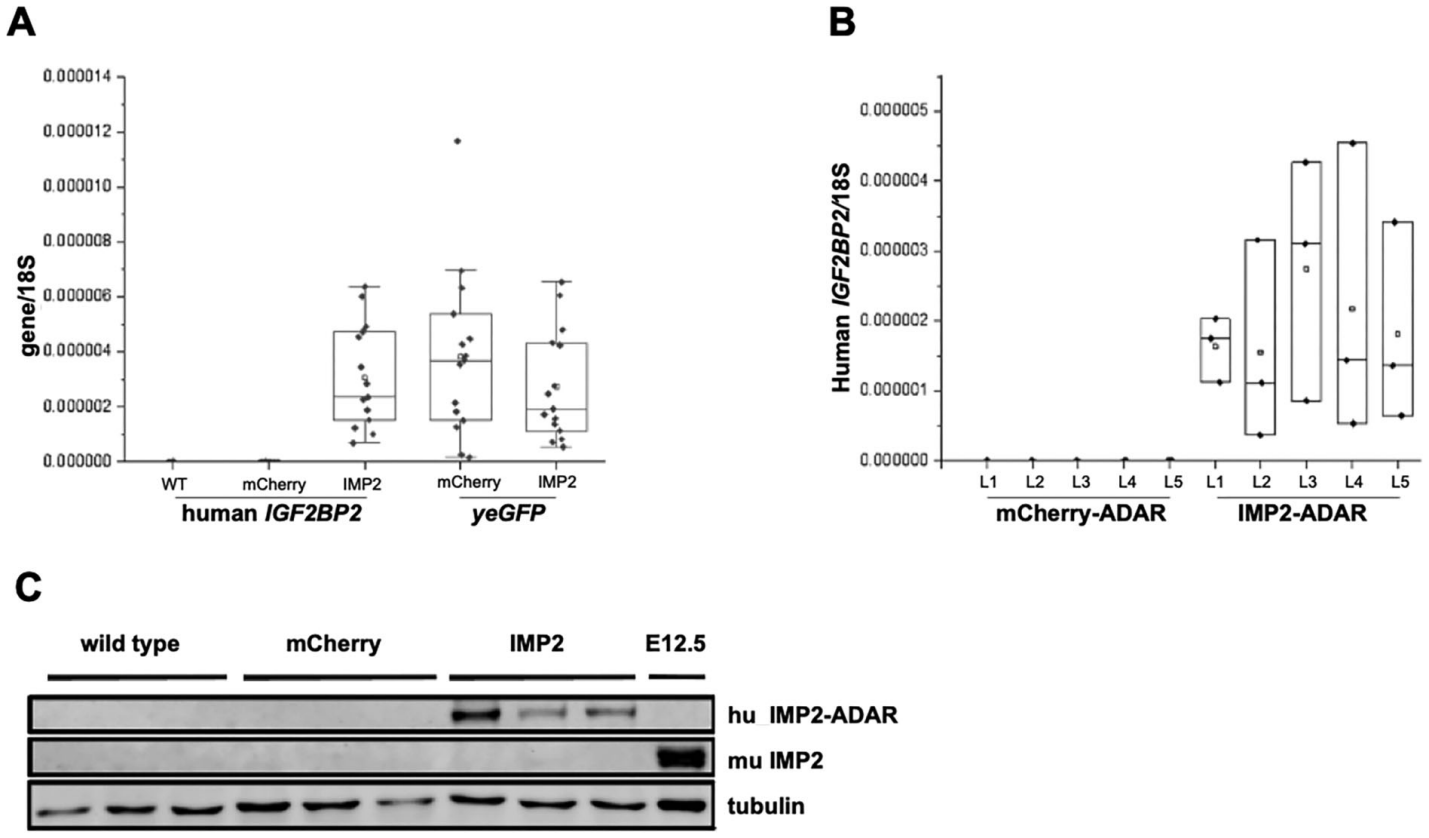
Control of plasmid DNA transfection in murine livers by quantitative RT-PCR analysis and western blot. (**A**) Overall expression of human IMP2 (*IGF2BP2*) and fluorescence tag yeGFP in mCherry–ADAR-injected control (mCherry–ADAR) and in IMP2–ADAR injected experimental mice compared to non-injected wild-type (WT) mice. Individual values (rhombi) are presented in a box plot with squares as mean values and whiskers as ±SEM. ***: *P* < .001 (One-way ANOVA). (**B**) Expression of IMP2 (*IGF2BP2*) in the individual liver lobules. L1: left lateral lobe; L2: left medial lobe; L3: right medial lobe; L4: right lateral lobe; L5: caudate lobe. Bars represent mean ± SEM and rhombi represent single liver lobules from each mouse, *n* = 3 (triplicates of each of the five liver lobules). In both analyses, *Rn18s* was used as the housekeeping gene. (**C**) Western blot analysis of L1: left lateral lobes from non-injected wild-type mice, mCherry–ADAR injected controls, and IMP2–ADAR-injected experimental mice showing protein expression of human and murine IMP2. Liver from day 12.5 embryonic mice was used as murine IMP2 positive control [7].

Different strategies were followed to ensure successful transfection and to determine the transfection efficiency in hepatocytes: quantitative RT-PCR analyses revealed highly abundant levels of human *IGF2BP2* in IMP2–ADAR-transfected livers (Fig. 1A), indicating a successful gene delivery. Comparable expression levels of the marker *yeGFP* in both mCherry–ADAR- and IMP2–ADAR-transfected mice indicate similar transfection efficiency in both groups (Fig. 1A). As expected, *IGF2BP2* expression was not detected in both control-injected and wild-type mice due to a missing CDS of human *IGF2BP2*/*IMP2*. To determine whether a particular liver lobule was favored using HGD, the lobule-specific expression of *IGF2BP2* was investigated (Fig. 1B). There was no statistically significant difference of *IGF2BP2* expression between the single liver lobules in IMP2–ADAR-transfected mice. In addition, western blot analysis clearly confirmed the expression of the IMP2–ADAR fusion protein at the predicted size of 113 kDa in livers of IMP2–ADAR-transfected mice, while it was absent in livers of wild-type and mCherry–ADAR-transfected mice. Murine IMP2 was not detectable in any of the 12–14-week-old mice while being present in embryonic mice (Fig. 1C).

### Identification of IMP2 binding targets by HyperTRIBE

As the number of identified IMP2 targets varied greatly across the 16 computational schemes utilized to combine A2G sites across replicates, the most robust and specific scheme was selected with respect to concordance between the resulting genes and genes with editing sites detected in MEFs by HyperTRIBE analysis (Supplementary Table S4), as well as DEGs identified by DESeq2 (Supplementary Table S5). Given that IMP2 naturally exhibits high expression levels only during embryonic development [6], the selection of a suitable cellular model for comparison was imperative. To identify genes that distinguish IMP2 from WT or mCherry samples, yet behave similarly within the two control groups, genes were gathered from WT versus IMP2 and mCherry versus IMP2 comparisons and subsequently, genes found in WT versus mCherry were removed from this group. Gene sets corrected in this manner are referred to as “background-corrected IMP2-specific genes” or labeled “WT/mCherry versus IMP2 - WT versus mCherry.” The same background correction procedure was applied to DEGs from DESeq2 to ensure that HyperTRIBE and DESeq2 results are comparable for concordance analysis (Supplementary Table S5).

Based on the selected replicate collapsing scheme detailed in Supplementary Information Section S1, IMP2 target genes were defined as those with A2G editing sites in their 3′/5′-UTRs/CDS that had transcript coverage higher than 1% and were identified in at least two out of three replicates. The background-corrected IMP2 target genes that resulted from this scheme exhibited the largest overlaps with those from HyperTRIBE analysis of MEF samples and DEGs from DESeq2, in comparison to other schemes (Supplementary Tables S3–S5 and  Supplementary Figs S8 and S9). The results that explain the selection of this collapsing scheme are summarized and discussed in the “Replicate collapsing scheme selection” section of Supplementary Information S1. Genes that were both significantly deregulated according to DESeq2 and contained HyperTRIBE-identified A2G sites, before and after background correction, are listed in Table 1.

**Table 1.** Genes with deregulated transcript levels and identified as IMP2 targets

Comparison	Deregulated IMP2 target genes	Gene count	Jaccard similarity (x1000) between IMP2 target genes and DEGs
WT versus IMP2	Calu, Ccdc90b, Cdadc1, Clta, Cped1, Cplane1, Creb1, Crem, Cyp2c38, Dck, Ddx11, Ddx58, Dhx58, Dlg1, Ecm1, Elmod3, Esam, Fktn, Gm20604, Grk6, Hck, Igtp, Macf1, Mad2l2, Me2, Met, Mff, Mmrn2, Mtif3, Mug1, Numb, Parp14, Pecam1, Plec, Ppil3, Rabep1, Reln, Retreg1, Rnf38, Slc66a2, Stx5a, Syt12, Taf6, Tardbp, Tdrd7, Tgtp1, Tmem62, Tnpo1, Trim39, Vps39, Wnk1	51	27.55
mCherry versus IMP2	Creb1, Crem, Fam219a, Fktn, Gm20604, Map7d1, Med16, Nfix, Prpf40b, Ptbp3, Rabep1, Smim14, Taf6, Tardbp, Tnpo1, Vps39	16	12.26
WT versus mCherry	Add3, Cdadc1, Cyp27a1, Dhx58, Dlg1, Dusp12, Fktn, Gm11837, Hsd3b5, Lrp11, Mmrn2, Oasl1, Pigw, Plec, Pnpt1, Prpf40b, Retreg1, Rnf38, Slfn4, Tdrd7, Ttbk2, Ubr5, Wnk1, Zfp708	24	17.32
Background-corrected IMP2 target genes	Agfg1, Ccdc90b, Creb1, Cyp2c38, Dck, Mad2l2, Med16, Rabep1, Taf6, Tardbp, Tmem62, Tnpo1, Trim39	13	12.62

Genes with A2G sites from HyperTRIBE analysis (using 1% average editing threshold, combined for mutual CDS/UTR across at least two replicates) and differential expression from DESeq2 analysis (|LFC| < 1, FDR-adjusted *P*-value <.05) are listed in the second column. The gene counts are reported for each pairwise comparison among WT, mCherry, and IMP2 samples and for “background-corrected IMP2 target genes:” genes identified in the comparison between any control and IMP2, but not in the WT versus mCherry comparison. The similarity between IMP2-specific genes and DEGs was measured using Jaccard Index x 1000.

### Profiling of identified editing sites

#### Functional enrichment analysis of IMP2 target genes and deregulated genes

Identifying the biological processes enriched among IMP2 target genes or control genes should help in better understanding how IMP2 targets may potentially impact cell function by altering the transcriptomic profile. Thus, GO enrichment analysis was performed for the sets of genes that contrasted control and IMP2 samples in the HyperTRIBE experiment and differential transcripts analysis.

**Figure 2.**
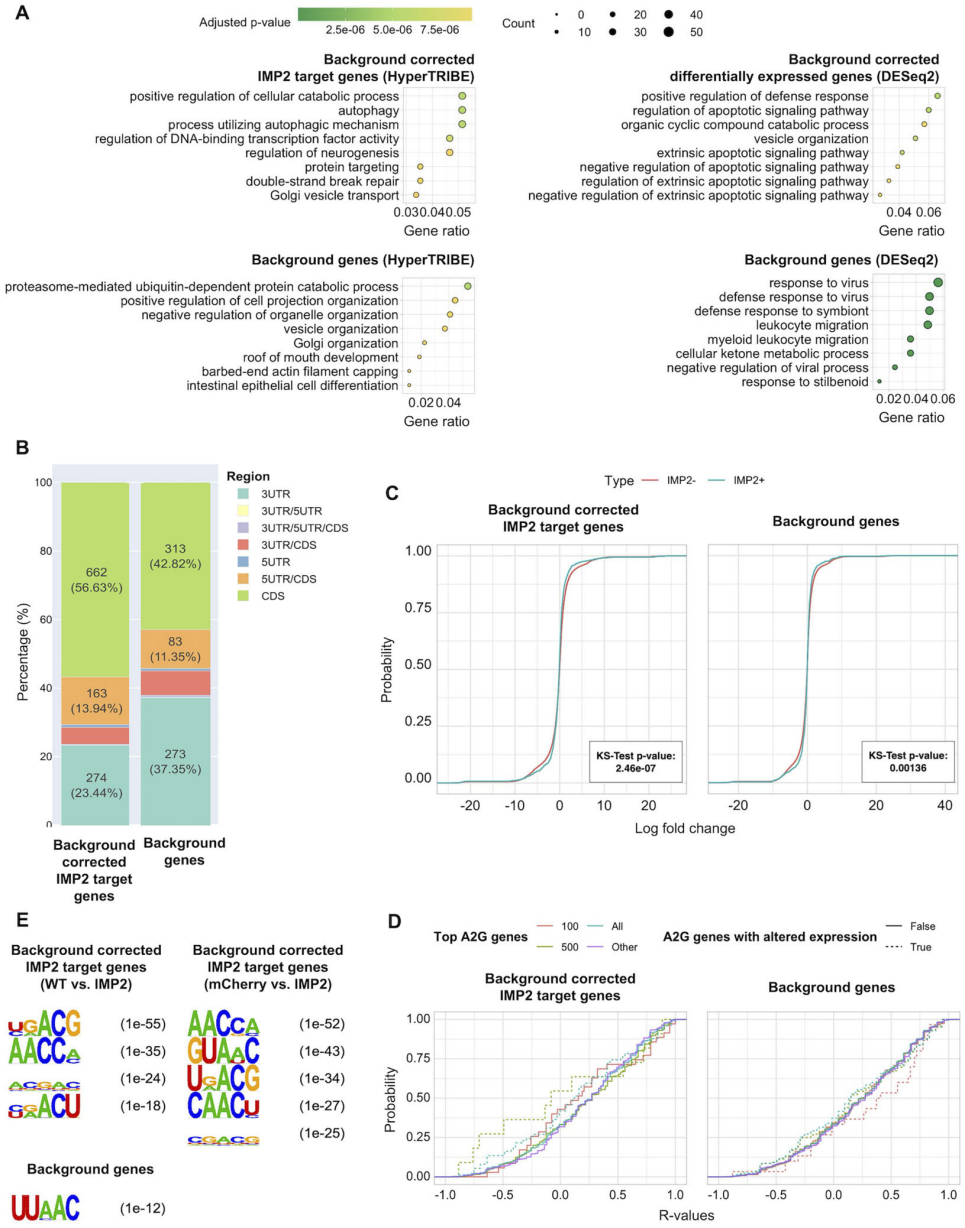
Profiling of background genes and background-corrected IMP2-specific target genes identified by HyperTRIBE. (**A**) Most significantly enriched biological process terms (FDR ≤ 0.05) from gene ontology enrichment analysis of IMP2 target genes (left panel) and DEGs (right panel). Enrichments were computed with the R package clusterProfiler. (**B**) Distribution of A2G sites remained for the selected replicate collapsing scheme (T = 1%, UNION, CDS/UTR). The regions containing A2G sites identified with at least 1% transcripts coverage (T = 1%) in CDS or 3′/5′-UTR in at least two out of three replicates (UNION) were annotated using the *M. musculus* reference genome *mm39*. (**C**) Cumulative distribution of LFC for IMP2 target genes. The DESeq2-computed LFCs that displayed changes in gene expression levels were plotted for genes with and without A2G sites separately, denoted by IMP2+ and IMP2− in blue and red, respectively. Kolmogorov–Smirnow tests were used to compare the cumulative distribution between any IMP2− and IMP2+ set of LFCs. (**D**) Cumulative distribution of Spearman correlation coefficients in expression level between IMP2 and other genes. Genes were categorized into four color-coded groups: all genes with A2G sites (All), top 100 and 500 A2G genes with the highest editing percentage (100 and 500, respectively), and genes with no A2G sites (Other). The distributions for A2G genes with deregulated expression identified by DESeq2, listed in Table 1 and Supplementary Table S6, are plotted in dashed lines. (**E**) *De novo* motif analysis and differential analysis of m6A-related motifs enriched in IMP2 binding regions using the HOMER Motif Discovery tool. *De novo* motif analysis was performed for sequences in a [−500 bp, +500 bp] region around each A2G site for each comparison group using a motif search window of size 200 bp. In differential motif analysis, all positions identified in the WT versus mCherry comparison (bottom left) were removed from the WT versus IMP2 (top left) and mCherry versus *IMP2* (top right) comparisons. Significantly enriched motifs (FDR ≤ 1e−10) are tabulated in Supplementary File S2. Only motifs similar to RAC/RACH/DRACG variants of the m6A consensus motifs and their adjusted *P*-values are shown here, such that D = A/G/U, R = A/G, and H = U/A/C. Full reports of these analyses for all pairwise comparisons are shown in the Supplementary Data such that Figs S11, S13, S14, S15, and S17 correspond to subfigures A–E, respectively. Background gene sets are labeled “WT versus mCherry” and background-corrected gene sets are termed “WT/mCherry versus IMP2 - WT versus mCherry.”

Several biological processes were revealed to be enriched in IMP2-bound control genes, in background-corrected genes (Fig. 2A, left side), and in deregulated genes (Fig. 2A, right side). After determining background-corrected IMP2-specific genes, it was revealed that background IMP2-bound mRNAs were enriched in catabolic processes and organelle/cellular organization (Fig. 2A, bottom left). For IMP2 target genes after background removal, the majority of catabolism-related terms were pruned, whereas autophagy terms were retained (Fig. 2A, top left). Meanwhile, background DEGs were most enriched in cellular defense mechanisms (Fig. 2A, bottom right). Interestingly, eliminating background DEGs resulted in the loss of most terms related to immune responses, while GO terms for apoptosis and catabolic process emerged with high frequency (Fig. 2A, top right). While IMP2 has been previously implied in both autophagy and apoptosis in the context of cancer progression [18, 50], the tight interconnection between these processes has been portrayed as sequential [51], whereby Wnt/β-catenin signaling plays an important role [52]. Additional results of gene ontology enrichment analysis for IMP2 target genes and DEGs of each pairwise comparison across three samples are shown in Supplementary Fig. S11.

**Figure 3.**
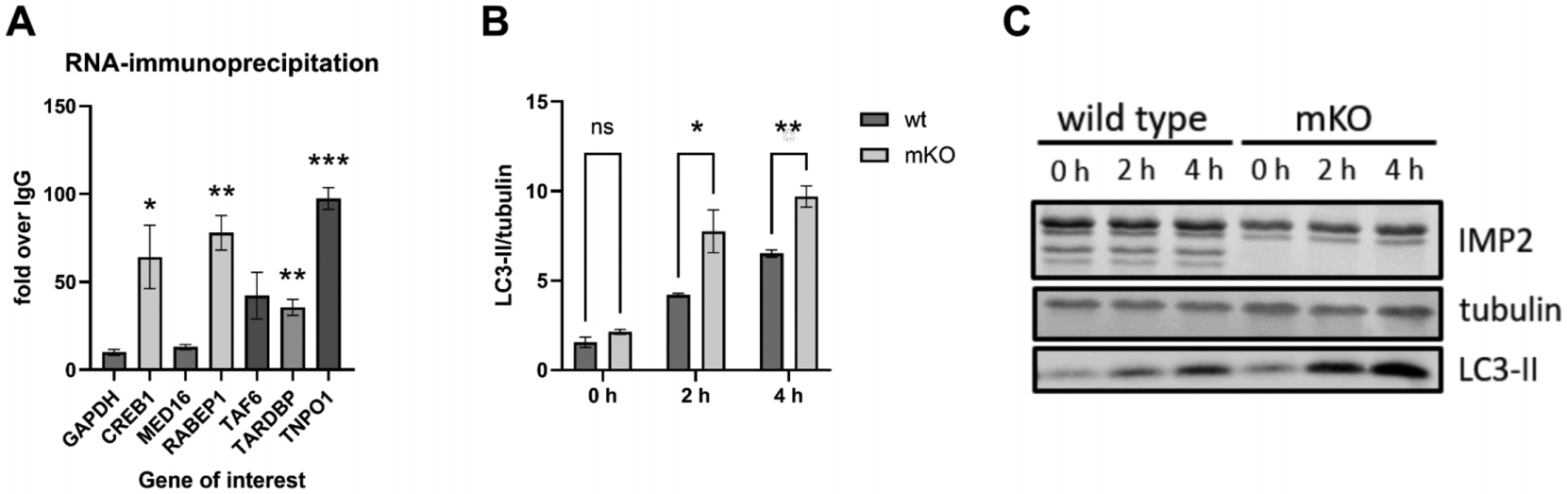
Experimental validation of TRIBE targets. (**A**) RIP analysis in Huh7 followed by qPCR of IMP2 targets. Expression was normalized to IgG control. GAPDH served as a negative control. Mean values ± SEM of *n* = 3 experiments are shown. The distribution of expression levels was initially subjected to a normal distribution curve, and subsequent statistical differences were calculated using a two-sample *t*-test. (B, C) Western blot analysis of IMP2 and its splice variants, as well as LC3-II in HepG2 wildtype and monoallelic IMP2 knockout cells, after treatment with 10 nM bafilomycin for 0–4 h. (**B**) Quantification of the LC3II/tubulin ratios. Data are expressed as mean ± SEM. Statistical differences were calculated using multiple unpaired *t*-tests (Bonferroni–Dunn method). *P < .05; **P < .01. (**C**) Representative images of three (*n* = 3) biological replicates are shown.

In order to experimentally validate the observed TRIBE targets, RIP of IMP2 was performed in the human hepatocyte cell line Huh7. *Creb1, Med16, Rabep1, Taf6, Tardbp*, and *Tnpo1* were extracted from Table 1 as most significant potential RNA targets. Indeed, RIP analysis confirmed binding of *Creb1, Rabep1, Tardbp*, and *Tnpo1* by IMP2. To further confirm the suggested effect of IMP2 on autophagy, LC3 western blot analysis was performed. While IMP2 proved highly abundant in wild-type cells, it was significantly downregulated in a previously published CRISPR/Cas9-generated *monoallelic IMP2 knockout* human hepatocyte cell line [10]. Indeed, after inducing inhibition of late-stage fusion of autophagolysosomes with bafilomycin, IMP2-reduced hepatocytes featured significantly higher levels of the autophagy marker LC3-II when compared to wild-type cells (Fig. 3).

#### Distribution of A2G sites in genes

Given that CDS and UTR were selected as regions to combine A2G sites present in at least two out of three replicates in each sample group, Fig. 2B shows the distribution of these sites in CDS, 3′UTR, and 5′UTR regions in both background and background-corrected IMP2-specific genes; Supplementary Fig. S13 shows the same results for each pairwise comparison across WT, mCherry, and IMP2 samples. As the *mm39* reference genome was used, which contains overlapping transcripts, some regions may simultaneously possess multiple labels. However, it was observed that very few regions were annotated both as 3′UTR and 5′UTR, thus the overall resulting statistics were not compromised. In each pairwise comparison between WT, mCherry, and IMP2 samples, most IMP2 targets were edited in their CDS (42.82%–48.49%) or in their 3′UTR (32.45%–37.35%) (Supplementary Fig. S13). When filtering out A2G sites that were detected in comparisons of the two controls (WT versus mCherry), the proportion of sites remarkably increased to 56.63% for CDS and decreased to 23.44% for 3′UTR (Fig. 2B). Meanwhile, only marginal changes were detected in the percentages of other annotated regions after this filtering step.

#### Effects of IMP2 binding on targeted transcripts

Previous studies showed that IMP2 binding tends to stabilize targeted mRNAs [12], prompting analysis of our own dataset to examine whether such a conclusion can also be derived. The full gene set was partitioned into two groups: genes with and without identified A2G editing sites, termed IMP2+ and IMP2−, respectively. After sorting, the cumulative distribution of their LFC values was plotted for all pairwise comparisons, as detailed in Supplementary Fig. S14. The LFC distributions of background genes and background-corrected IMP2-related genes are shown in Fig. 2C. A Kolmogorov–Smirnov test was conducted to test whether there exists a significant difference in the LFC distributions across experimental groups. In all cases, it was revealed that genes with A2G sites showed a significantly lower LFC than genes without A2G sites, suggesting mRNA stabilization in IMP2-targets, which corroborates previous findings (Fig. 2C and Supplementary Fig. S14).

Considering this new evidence, it was speculated that there may exist a negative correlation between expression levels of IMP2 and IMP2 target genes. Hence, the Spearman correlation coefficient was computed between each IMP2 target gene and IMP2 itself. Similarly to the LFC distribution, the cumulative distribution of Spearman coefficients is shown for the same four comparisons in Supplementary Fig. S15, with Fig. 2D contrasting the correlation of background genes (WT versus mCherry) with the correlation of background-corrected IMP2-specific genes (WT/mCherry versus IMP2 - WT versus mCherry). To this aim, IMP2 target genes were split into two groups on the basis of being deregulated (|LFC| > 1, FDR < 0.05) or not, followed by a ranking by LFC values into top 100 and 500 genes. Generally, deregulated IMP2 targets differ noticeably from deregulated non-targets, as well as from IMP2 targets that are not deregulated; see Fig. 2D and Supplementary Fig. S15. Referring to Fig. 2D, the distribution of deregulated top 100 IMP2 targets, illustrated by the red dashed curve, features a larger anticorrelation than non-targets, depicted by the solid purple curve. For the deregulated IMP2 targets ranked in and under the top 500 targets, (plotted by the green dashed and blue dashed curves, respectively), this effect is blunted in all pairwise comparisons of Fig. 2D. As observed in panels (A)–(C) the effect is particularly noticeable when background genes were omitted from identified IMP2 targets.

Additionally, the same analysis was performed on four public datasets retrieved from GEO NIH, with the following series numbers: GSE14520 (cohorts 1 and 2), GSE57957, and GSE54236 (Supplementary Fig. S16). For dataset GSE14520, data from both cohorts demonstrate higher anticorrelation in top 100 A2G genes when compared to genes without A2G sites, depicted, respectively, by the red and purple curves in Supplementary Fig. S16B and C for the first three groups. This is not the case for GSE57957 and GSE54236, where the majority of A2G-gene expression correlates positively to IMP2 expression. For all datasets, correcting IMP2 targets revealed A2G genes with weaker correlation to IMP2. Thus, pertaining to the largest dataset, GSE14520, this analysis conducted on human liver cancer tissues confirmed anticorrelation of IMP2 targets, further indicating mRNA target stabilization by IMP2.

#### Motif analysis of IMP2 binding targets

To identify molecular details that suggest how IMP2 may stabilize mRNA transcripts, motif enrichment analysis was performed in regions of 500 bp up- and downstream from an A2G site. The enriched motifs from this analysis with *P*-values <1e−10 are listed in Supplementary File S2. In total, the HOMER software identified 27 *de novo* motifs to be enriched around A2G sites, as detected by HyperTRIBE for WT versus IMP2, mCherry versus IMP2, and WT versus mCherry. The number of significant hits was lowest for the WT versus mCherry group. While more motifs were enriched in the WT versus IMP2 and mCherry versus IMP2 groups, these two sets of results shared only one common motif: UUACC(UUACC). Subsequently, sequences of genes that have edited A2G sites within the WT versus mCherry group were extracted from the WT versus IMP2 and mCherry versus IMP2 groups as background sequences for differential motif analysis by HOMER (Fig. 2E). After background removal, the similarities between the two groups grew more pronounced, with 13 common motifs among 34 distinct enriched motifs. As shown in Supplementary Fig. S14 A–C, out of these 13 motifs, 4 are similar to RAC/RACH/DRACG variants of m6A consensus motifs [53–55]: UGACG, AACCA, AACCA, and UGACG. In total, differential motif analysis identified 7 out of 34 motifs that resembled m6A motif variants (Fig. 2E).

## Discussion

Currently, RBP target discovery largely relies on CLIP-based methods that feature high specificity and robustness. However, high throughput immunoprecipitation experiments require substantial amounts of input materials and are limited by the efficiency of RBP-protein crosslinking, as well as by the sensitivity of transcripts to enzymatic activity in the subsequent RNase digestion step [56]. To surmount these shortcomings, the HyperTRIBE protocol utilizes a fusion construct between an RBP of interest and the enzyme ADAR. In subsequent sequencing, it detects readouts with modified ADAR-deaminated adenosine-to-inosine bases (A2G sites), revealing RBP-binding sites [28]. In contemporary literature, targets identified by HyperTRIBE were found to be consistent with those detected by CLIP [28]. Yet, HyperTRIBE features reduced experimental cost and complexity, lower sequencing depth bias, and results that are not affected by crosslinking efficiency. In this study, HyperTRIBE was adapted to identify IGF2BP2-binding targets in mouse hepatocytes in their tissue environment. This was facilitated by hepatocyte-specific transfection with the experimental plasmid IGF2BP2–ADAR, as well as a control mCherry–ADAR plasmid, *in vivo*.

In the first computational step, A2G sites detected in the sequenced reads were mapped to the mouse reference genome and results for IMP2 samples were contrasted against those from mCherry and wild-type (WT) samples. While mCherry samples served as positive controls for transfection in hepatocytes, WT samples were ascribed as negative controls for both transfection and the IMP2 binding assay. As HyperTRIBE uses ADAR with an E488Q hyperactive mutation that reduces editing biases in certain sequences and structures as compared to the TRIBE method, a certain degree of biases may still exist and can be detected when comparing any sample to WT samples [28, 43]. Hence, A2G sites present in IMP2 that were not in either of the two control comparisons, WT versus IMP2 and mCherry versus IMP2, along with sites present in none of the controls, WT/mCherry versus IMP2 - WT versus mCherry, were inspected. Genes with A2G sites in the latter group are referred to as “background-corrected IMP2-specific genes,” as they do not contain background genes with A2G sites that distinguish only control samples in the WT versus mCherry comparison. The selection of background gene sets, to account for biases from ADAR background activities, was discussed in the “Background activity of ADAR” section of Supplementary Information S2.

First, it should be noted that edited A2G sites should not be considered as IMP2 binding sites, as ADAR does not compete with RBP-binding spots, suggesting that defining IMP2 targets solely based on individual A2G sites without considering proximal regions may not yield adequate and accurate conclusions. Our decision of considering entire CDS and UTR regions is reinforced by previous findings that RBP-binding modes are highly diverse, with preference to certain sequences, such as GC-rich sequences, to specific structures, such as loop regions, and to genomic regions, as observed with UTR. This generates a vast variety of possibilities for unconventional RBP-binding domains [57]. For each edited site, HyperTRIBE quantifies the editing percentage based on read counts in IMP2 samples and background samples, implying that selecting significant A2G sites based on editing percentage is greatly impacted by how one combines results across replicates in IMP2 or background samples. In fact, the authors of the HyperTRIBE protocol recommended selecting only A2G sites shared across all replicates to ascertain a high level of confidence, or to use a high threshold of 10% to define the significant editing percentage [28]. Here, the outcomes of different A2G site selection schemes across replicates were compared, as well as the defining of IMP2 targets based on ADAR-edited sites or regions. Details are given in the “replicate-collapsing schemes” section.

The schemes were assessed by their statistical power, their stability in detecting IMP2 targets, by their concordance to the results from HyperTRIBE experiments on MEFs, and by their association with deregulated genes, respectively. The outcomes collectively suggested that CDS, 3′UTR, and 5′UTR are appropriate genomic spans for grouping A2G sites into “edited regions,” given that they contain any edited sites in two out of three replicates with 1% editing percentage across all transcripts (1%-UNION-CDS/UTR). The selected scheme preserves reproducibility by requiring the edited sites to be found across different replicates while relaxing the constraint for exact site overlap, which enables it to generate sufficient results for further analyses (Supplementary Fig. S8 and Supplementary Tables S3 and  S6). Additionally, selecting CDS/UTR as target-defining regions demonstrated considerable robustness, as these were the only regions that detected the same IMP2 targets (Supplementary Fig. S9). Annotating A2G sites to CDS/UTR regions also shifts the focus to regions previously identified as IGF2BP2 or ADAR binding hot spots [1, 58]. As shown in Supplementary Fig. S13, in all pairwise comparisons among IMP2, WT, and mCherry samples, IMP2 showed strong binding preference for regions within 3′UTR, at 33.38%–37.35%, and CDS, at 42.82%–48.49%. This finding concurs with previous studies that have unveiled binding preferences of IMP2 [58, 59]. Interestingly, in the set of background-corrected IMP2 target genes, considerably fewer IMP2 targets were found in 3′UTR, at a frequency of 23.44%, while more were detected in CDS, at 56.63% (Fig. 2B).

Differential gene expression analysis derived with DESeq2 showed that more genes were upregulated in mCherry or IMP2 samples, as opposed to those in WT (Supplementary Figs S6 and S7). Raw expression levels and DEGs proved more similar between mCherry and IMP2 groups than those within the WT group (Supplementary Figs S3–S7). As a result, it is evident that the deregulated genes in the WT versus IMP2 and WT versus mCherry comparisons share annotations to more similar and more significant biological processes with each other than with those in the mCherry versus IMP2 comparison (Supplementary Fig. S11E–G). Notably, this is not the case when referring to the analysis of IMP2 targets with HyperTRIBE. The enriched biological functions for IMP2 targets share more overlap between WT versus IMP2 and mCherry versus IMP2 than with WT versus mCherry, specifically for autophagy and regulation of catabolic processes (Supplementary Fig. S11A–C). Additionally, only a small subset of IMP2 targets that were also deregulated was found (Supplementary Tables S4 and S5). This discrepancy between DESeq2 and HyperTRIBE analysis implies that IMP2 can affect the transcriptome without drastically altering the expression of target genes, emphasizing the complex nature of RBP activity in cells. However, in both DESeq2 and HyperTRIBE analyses, background correction on the set of identified deregulated genes or on IMP2-specific genes revealed genes enriched with cellular catabolic processes, and either autophagy (HyperTRIBE) or apoptosis (DESeq2) (Fig. 2A, top). Autophagy and apoptosis are two deeply interwoven processes that regulate cell death and survival by mediating cellular and organismic homeostasis in response to stressors [60]. In particular, it has been reported that genes involved in regulation of autophagy overlap with apoptosis-associated genes [61], which supports the assumption that IMP2-bound genes may modulate immune responses and apoptotic activation through autophagy.

Results from RIP assays have confirmed binding of IMP2 to autophagy-associated transcripts and increased LC3-II levels upon IMP2 knockdown in an HCC model cell line (Fig. 3A and B), indicating that IMP2 affects autophagy. A potential link between IMP2 and autophagy could be the ability of IMP2 to induce steatosis in mice [15, 17, 62], which is strongly associated with dysfunctional autophagy [63]. Although the complex role of autophagy in lipid metabolism in steatosis models is yet to be fully unraveled, literature suggests a negative correlation between autophagy and steatosis [63]. Moreover, it has been reported that modulated endogenous cholesterol synthesis spurred by transgenic overexpression of IMP2 in mouse liver activates NF-κB transcription factors, as well as several of their targets [17]. NF-κB is bidirectionally linked to the regulation of autophagy in a variety of physiological and pathological contexts [64–66] and may also serve as an indirect mediator between IMP2 and autophagy. Furthermore, Li *et al*. [67] reported decreased p62/SQSTM1 levels and an increased LC3-II/LC3-I ratio in glioma cells upon IMP2 knockdown, supporting our findings in hepatocellular carcinoma cells. IMP2 was also shown to promote the progression of breast cancer by degrading the RNA transcript encoding a subunit of v-ATPase [19, 68], which serves as a major regulator of LC3 [69]. In contrast, Han *et al*. [70] showed that IMP2 increases the stability of the lncRNA *MALAT1*, which upregulates the autophagy-related gene *ATG12*, thereby activating autophagy in a non-small cell lung cancer model. Although the exact mechanism of action remains unclear, we suggest that IMP2 deregulation may also impact autophagy in the liver.

As previously reported, IMP2 is linked to mRNA stabilization in colorectal cancer [12, 19]. In our study, we also found IMP2 targets to be more stabilized by contrasting the changes in expression between IMP2-target and non-target genes (Supplementary Figs S13 and S15, and Fig. 3C and D). Although DESeq2 showed that gene expression levels in mCherry and IMP2 were more similar than in WT (Supplementary Figs S6 and S7), the cumulative distributions of the LFC derived from DESeq2 analysis, separated on the basis of whether a gene is an IMP2-target or not, show more significant differences when comparing mCherry or WT against IMP2 (Supplementary Fig. S14 and Fig. 2C). Additionally, IMP2 targets with strong deregulation show anticorrelation to IMP2 (Supplementary Fig. S15), especially in the mCherry versus IMP2 comparison, where ~60% of most frequently edited genes display a weak-to-moderate positive correlation to IMP2. While these findings suggest IMP2’s potential to destabilize IMP2 target genes, a portion of deregulated targets are observed with a higher correlation to IMP2 instead (depicted by the green and blue dashed lines in Supplementary Fig. 15B). This effect, though unobservable when IMP2 targets were background-corrected in Fig. 2D, suggests that studies on specific target genes for IMP2 are necessary to further understand the stabilizing effects of IMP2.

IMP2 has been reported as an essential m6A-reader and prefers binding to DRACH, m6A’s consensus motif in *M. musculus*, as well as its variants: RRACH, URACH, RACH, RRAC, DRAY, RAC, and DRACG, such that D = A/G/U, R = A/G, H = U/A/C, and Y = U/C on target mRNAs [53–55]. In our results, targeted transcripts are shown to be more resistant to changes in expression levels, suggesting a connection to IMP2-directed stabilization, e.g. due to initiating m6a modification. Thus, *de novo* motif analysis was performed to discover IMP2’s binding preference within +/−500 bp of an A2G site and its potential link to m6A motifs. Remarkably, UGACG, a motif similar to DRACG, was found recurrently around edited sites in the *de novo* analysis of WT versus IMP2 and in both differential motif analyses with top enrichment, for background-corrected WT versus IMP2 and mCherry versus IMP2 genes. Out of seven common motifs between corrected results for WT versus IMP2 and mCherry versus IMP2, four motifs were found to contain or resemble RAC/RACH/DRACG variants (Fig. 2E, top row). The results suggest that IMP2-targeted mRNAs may have been stabilized through IMP2’s preferential binding to m6A modification sites.

In summary, our study demonstrates successful *in vivo* adaptation of the HyperTRIBE protocol to identify IMP2 binding targets in mouse hepatocytes. By focusing on CDS and UTR regions, reproducibility and robustness of target identification was ensured, and our results were aligned with known IMP2 binding sequence preferences. Furthermore, our results demonstrated a direct interaction of IMP2 with transcripts involved in autophagy. So far, mainly target-stabilizing effects have been described for IMP2, urging further investigation of its destabilizing effects to clarify their functional implications.

## Supplementary Material

ugag034_Supplemental_Files

## Data Availability

The codes for data retrieval, data processing, and all analysis are made available on Zenodo (https://doi.org/10.5281/zenodo.14627021). MEF sequencing data are available on GEO with the accession number GSE260682. Sequencing data of mouse hepatocytes are available in the ArrayExpress database (http://www.ebi.ac.uk/arrayexpress) under accession number E-MTAB-14301.
