# Supplementary material for: HyperTRIBE identifies hepatic IGF2BP2/IMP2 targets in vivo and links IMP2 to autophagy
Source: NAR Mol Med. 2026 Jun 24;3(3):ugag034. doi: 10.1093/narmme/ugag034 (PMC13335485; doi:10.1093/narmme/ugag034)
Supplement: ugag034_Supplemental_Files [file ugag034_supplemental_files.zip › Supplementary Data_revision2_clean.pdf]

HyperTRIBE identifies IGF2BP2/IMP2 targets in vivo and links IMP2 to autophagy

Supplementary Data

Hoang Thu Trang Do[1], Simon Both[2], Tarek Kröhler[2], Marcello Pirritano[3], Elien Van Wonterghem[4], Sören Franzenburg[5], Emadeldin M. Ibrahim[1], Martin Simon[3], Jeetayu Biswas[7], Volkhard Helms[1,10*], Sonja M. Kessler[2, 6, 8], Alexandra K. Kiemer[2, 9,10,*]

[1] Center for Bioinformatics, Saarland University, Saarbrücken, 66123, Germany
[2] Saarland University, Department of Pharmacy, Pharmaceutical Biology, Saarbrücken, 66123, Germany
[3] Molecular Cell Biology and Microbiology, School for Mathematics and Natural Sciences, University of Wuppertal, Wuppertal, 42119, Germany
[4] VIB Center for Inflammation Research, Zwijnaarde, 9052, Belgium
[5] Institute of Clinical Molecular Biology, Kiel University, Kiel, 24105, Germany
[6] Institute of Pharmacy, Experimental Pharmacology for Natural Sciences, Martin Luther University Halle-Wittenberg, Halle, 06120, Germany
[7] Department of Medicine, Department of Molecular Pharmacology, Memorial Sloan Kettering Cancer Center, New York, NY 10065, USA
[8] Halle Research Centre for Drug Therapy (HRCDT), Halle, 06120, Germany
[9] Centre for Gender-Specific Biology and Medicine (CGBM), Saarland University, Saarbrücken, 66123, Germany
[10] PharmaScienceHub (PSH), Saarland University, 66123, Saarbrücken, Germany.

* Correspondence: pharm.bio.kiemer@uni-saarland.de and volkhard.helms@bioinformatik.uni-saarland.de

Supplementary Information

1. Replicate collapsing scheme selection

Summary: We established and applied 16 different schemes to combine A2G sites identified in different pairs of replicates when comparing two samples. As mentioned in Methods section (Supplementary Figure S3), the schemes are combinations of two editing thresholds used for coverage-based filtering of A2G sites (1% and 5%), four gene regions in which A2G sites should be combined (editing sites, 10bp windows, CDS/UTR, full transcript) and two ways of combining A2G sites (UNION: requires A2G sites to occur in at least two out of three replicates; INTERSECT: requires A2G sites to occur in all three replicates). The examined schemes resulted in greatly varying number of identified IMP2 targets (Supplementary Table S3); Thus, we compared the resulting genes to with editing sites detected by HyperTRIBE in mouse embryonic fibroblasts, and to genes detected by DESeq2 to be differentially expressed (Supplementary Information section 1.1 and 1.2, respectively). The largest overlap and highest robustness were obtained when using the **1% average editing threshold** and requiring detection of A2G sites in **3'UTR, 5'UTR or CDS** in **at least two out of three replicates** (Supplementary Tables S3-S4). Hence, we used this replicate collapsing scheme for the downstream gene set enrichment analysis and when discussing the biological relevance of our results.

1. *Overlap between HyperTRIBE results in mouse liver and in mouse embryonic fibroblast.*

We measured the overlap of gene sets reported by HyperTRIBE in murine liver and in MEF samples either as count of mutually found genes, the percentage of mutual genes among the results, or by the Jaccard index between the two gene sets. These results are shown in Supplementary Table S4.

Similar to the number of genes containing A2G sites, the overlap between mouse liver and MEF samples was larger when we compared larger genome spans, lower average editing percentage or fewer required replicates. The overlap was quite small when using a 5% threshold for the average editing percentage (Supplementary Table S4). Thus, we selected the results from the **1% threshold** to ensure better interpretability and robustness. On the other hand, filtering genes with A2G sites belonging to the same 10 bp window or CDS/UTRs yielded the highest percentage of mutual edited genes between mouse liver and MEF samples for the comparisons between any control and IMP2 (61.27% and 60.59% for 10 bp window and CDS/UTRs, respectively, for *IMP2 vs. WT* comparison and 60.42% and 59.98% for *IMP2 vs. mCherry* comparison) (Supplementary Table S4). Interestingly, although the number of mutual genes almost doubled when we expanded the editing regions from CDS/UTRs to full transcripts, the percentage of mutual genes in fact decreased, indicating a lower sensitivity.

2. *Overlap between HyperTRIBE results and DESeq2 differentially expressed genes.*

Next, we compared the editing sites in mouse liver detected by HyperTRIBE genes against the DEGs reported to be differentially expressed by DESeq2. Supplementary Table S5 lists Jaccard indices measured for the four editing sites/regions, where A2G sites were identified based on at least **1% average editing coverage** and in **at least two replicates**, as reasoned above. As expected, the Jaccard similarity increased when considering larger editing regions (Supplementary Table S5, columns A-D), but not as strongly as the increase in the numbers of detected genes having A2G editing sites (Supplementary Table S3).

Since we are interested in genes which distinguish IMP2 samples from WT or from mCherry but behave similarly for the two controls, we opted for the genomic span that could filter "control genes" most effectively. To this aim, we first gathered the genes from *WT vs. IMP2* and *mCherry vs. IMP2* comparisons (Supplementary Table S5, column D) and subsequently removed these genes from the comparison between control samples (Supplementary Table S5, column E). The fraction of genes that do not occur in the merged *WT vs. mCherry* result is reported in Supplementary Table S5, column F.

Although the number of A2G genes with altered expression increased 2-fold when using transcripts instead of CDS/UTR regions (26 and 13, respectively), the fraction of IMP2-related genes did not further decrease (both 0.19, Supplementary Table S5, column F). Additionally, all IMP2-related genes from the CDS/UTR-based scheme were also identified in all other schemes, whereas 13 out of 26 genes from the transcript-based scheme were not shared by any other scheme (Supplementary Figure S9). In general, aggregating all A2G sites in one CDS/UTR seems preferable as this resulted in a relatively large overlap between the HyperTRIBE and DEG gene sets without losing sensitivity and robustness in comparison to replicate collapsing-schemes using 10bp window or transcript. The genes that are both significantly deregulated according to DESeq2 and contain HyperTRIBE-identified A2G sites are listed in Supplementary Table S6.

2. **Background activity of ADAR**

Summary: We used two control samples in the experiment, including mCherry samples as positive control for transfection in hepatocytes and WT samples as negative controls for both transfection and IMP2 binding assay. Comparing IMP2 samples to either of these controls reveals IMP2 binding sites and regions through detecting A2G editing sites. As HyperTRIBE uses ADAR with a E488Q hyperactive mutation that reduces editing biases in certain sequences and structures as compared to TRIBE method, a certain degree of biases may still exist and can be detected when comparing any sample to WT samples (28, 43). Thus, we investigated the extent by which the three samples WT, mCherry and IMP2 differ from each other as means to measure ADAR background activities that might lead to editing biases and relevant changes in the transcriptomes. Surprisingly, the pairwise comparisons of HyperTRIBE results showed high consistency in detected A2G sites between WT and mCherry against IMP2 (Supplementary Information section 2.2), mCherry shared more similarity in transcriptomic profile with IMP2 than with WT (Supplementary Information section 2.1). Thus, to remove ADAR's background activity that might result in incorrectly identified editing sites, we performed a background correction for the set of IMP2 target genes identified in any comparison that IMP2 samples were involved in. We defined "background genes" as genes with A2G sites from *WT vs. mCherry* comparison and subtracted these from genes with A2G sites from *WT vs. IMP2* and *mCherry vs. IMP2* comparisons. For the sets of genes with differential expression, the same background-correction procedure was applied.

1. *HyperTRIBE's IMP2 target genes* - Supplementary Figure S11-S12, Supplementary Table S3-S4

To visualize the count distribution of editing sites per gene and to analyze whether the choice of control would affect the identification of editing sites, Supplementary Figure S12 plots counts of editing sites identified in different comparisons. Supplementary Figure S12C shows that both WT and mCherry identified highly consistent A2G numbers against IMP2 and can hence both serve as controls for identifying A2G sites in IMP2 samples. The genes identified in WT vs. mCherry generally had fewer A2G sites than in the comparisons WT vs. mCherry or in WT vs. IMP2 (Supplementary Figure S12A-B).

Supplementary Figure S11 lists the biological processes that were enriched in IMP2-bound genes and in deregulated genes, respectively. IMP2-bound mRNAs were enriched in catabolic processes, autophagy and cellular organization (Supplementary Figure S11A-C). In the last comparison (Supplementary Figure S11D), we removed control genes which belong to the *WT vs. mCherry* comparison. Then, the majority of catabolism-related terms were pruned, whereas autophagy terms were retained.

2. *DESeq2's differentially expressed genes - Supplementary Figure S4-S7, S10, Supplementary Table S7*

First, the homogeneity of each sample group, i.e., WT, mCherry, and IMP2, was assessed by principal component analysis (PCA). This showed that 97.38% of the total variance in the replicates is captured by the three first principal components (Supplementary Figure S4A). The three WT samples revealed the largest variation and were placed separate from the other samples. Along the fourth principal component, mCherry and IMP2 samples then show a split as well (Supplementary Figure S4B). The results of PCA analysis were consistent with correlation analysis of the raw read counts with Salmon using either Pearson or Spearman correlation (Supplementary Figure S5). Using DESeq2, we normalized and log-transformed the gene expression data in a multifactorial design, whereby one sample group was compared to one of the two other sample groups. Heatmaps of the differentially expressed genes then showed high agreement between the replicates of a sample group (Supplementary Figure S6). MA plots generated by DESeq2 revealed a symmetric distribution of transformed counts (Supplementary Figure S7).

The comparisons between *WT vs. IMP2*, *mCherry vs. IMP2*, and *WT vs. mCherry*, yielded 2252, 860, and 2068 transcripts belonging to 920, 264, and 832 differentially expressed genes identified by DESeq2, respectively ($|LFC| < 1$, FDR-adjusted p-value < 0.05). The Jaccard index of the sets of DEGs between WT and IMP2 and between WT and mCherry shows a good overlap of 0.49, Supplementary Table S7), which is also reflected in the heatmaps for those comparisons in Supplementary Figures S5A and C. On the other hand, the DEGs from the pair *mCherry vs. IMP2* (Supplementary Figure S6B) share little similarity to those from the other two comparisons (Jaccard indices of 0.13 and 0.14 for *mCherry vs. IMP2* and *WT vs. IMP2* in Supplementary Table S7, respectively).

While there are large overlaps in enriched gene ontology terms for IMP2 target genes from WT/mCherry to IMP2 comparisons, enriched terms for DEGs from mCherry/IMP2 to WT comparisons are more similar. DEGs from comparing mCherry/IMP2 to WT were enriched in cellular defense mechanisms (Supplementary Figure S11E and G). Notably, the enriched biological processes from mCherry vs. IMP2 DEGs, most of them being related to DNA and RNA processing (Supplementary Figure S11F), differ considerably from the DEGs derived from any comparison against the WT group (Supplementary Figure S11E and G).

Supplementary Figures

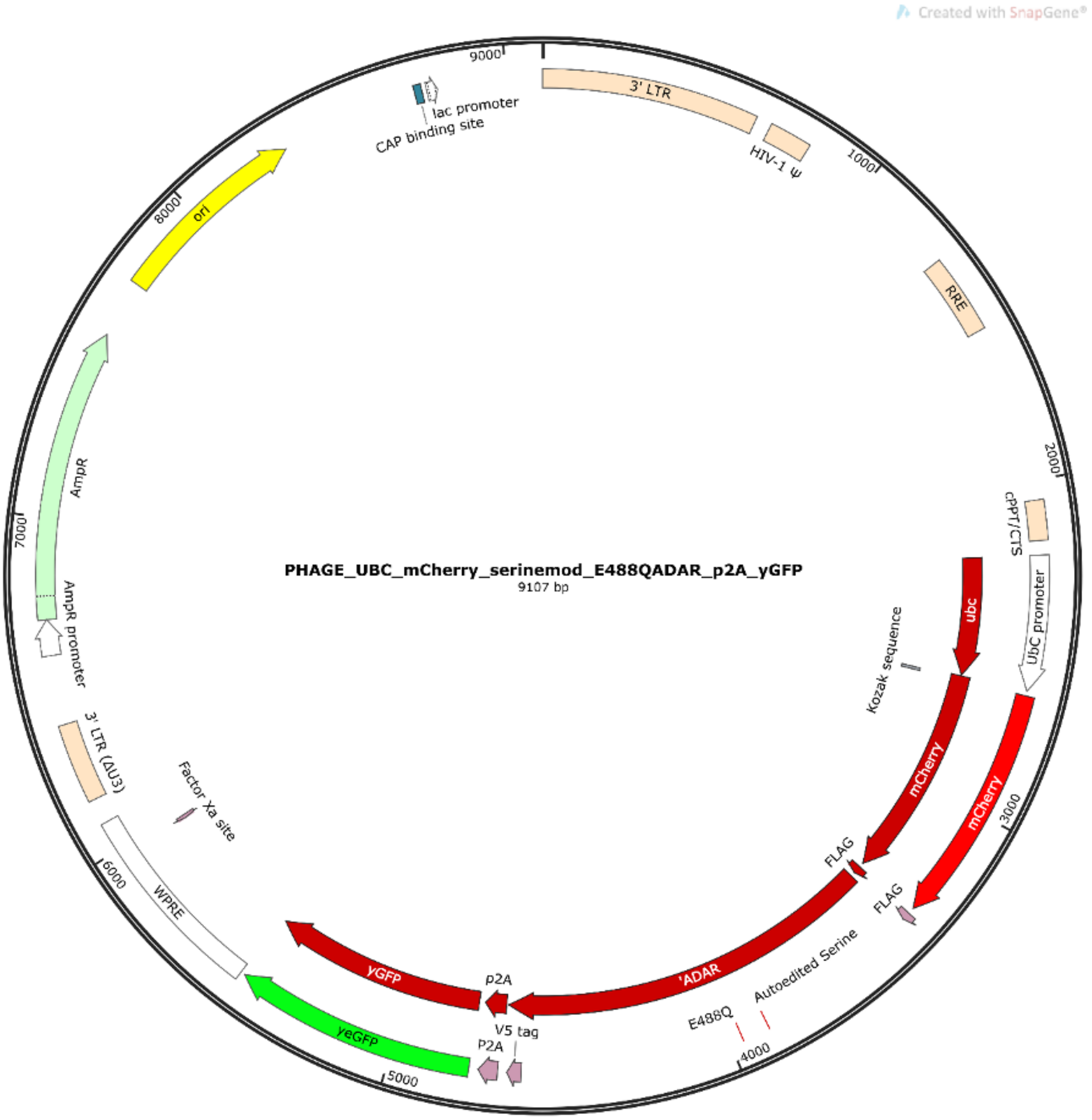

Supplementary Figure S1. A. PHAGE_UBC_mCherry_serinemod_E488QADAR_p2A_yGFP. Control plasmid “mCherry-ADAR” harboring a Ubiquitin C promoter (UbC), followed by the sequence of a fusion protein composed of mCherry with a FLAG-tag, ADAR, the self-cleaving peptide P2A, and a modified GFP (yGFP).

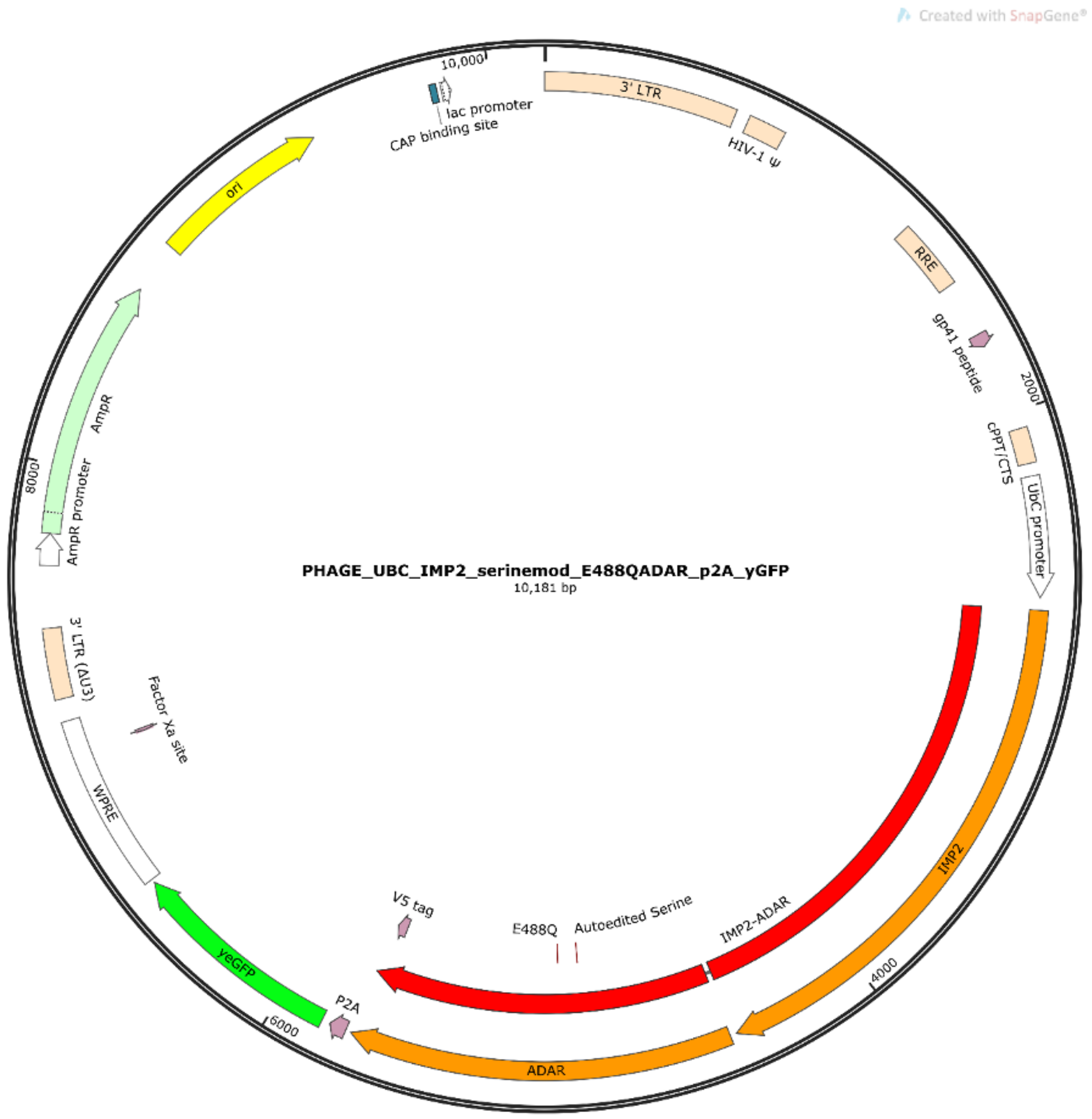

Supplementary Figure S1. B. PHAGE_UBC_IMP2_serinemod_E488QADAR_p2A_yGFP. Experimental plasmid “IMP2-ADAR” harboring a Ubiquitin C promoter (UbC), followed by the sequence of a fusion protein composed of IMP1, ADAR, the self-cleaving peptide P2A, and a modified GFP (yGFP).

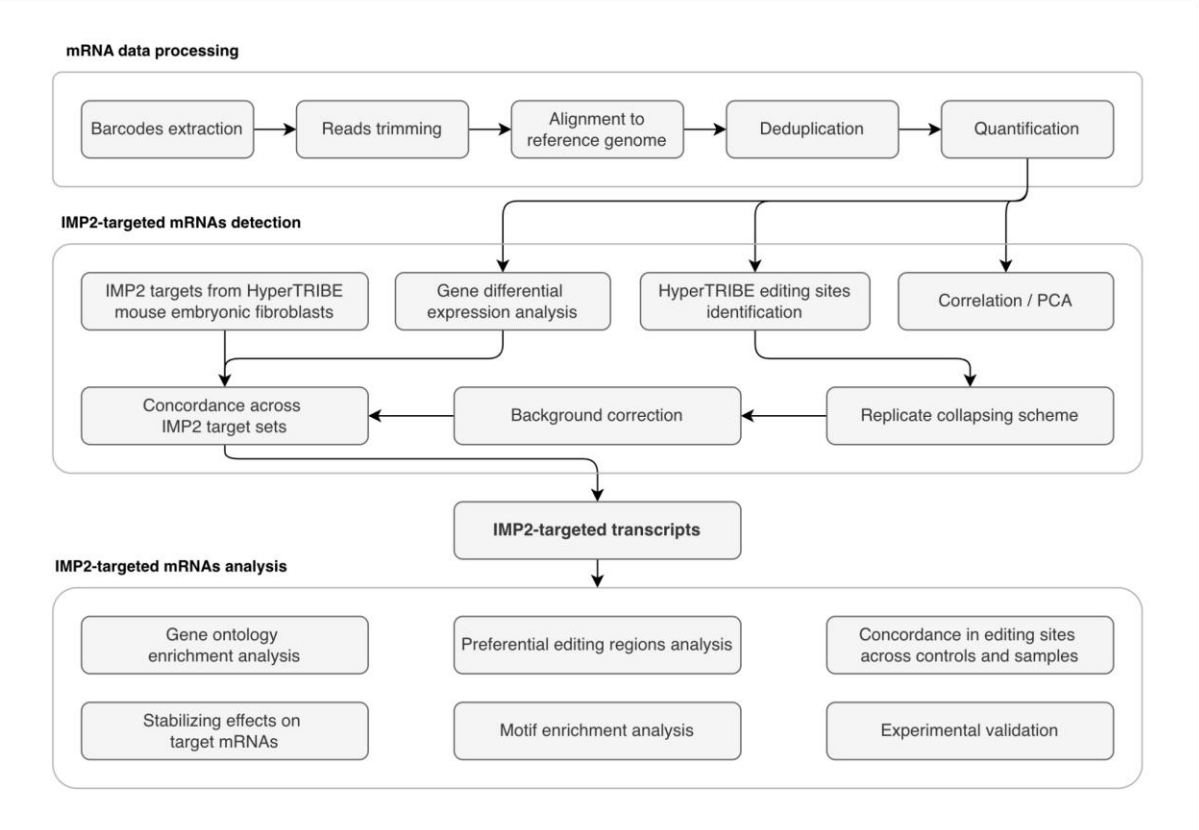

Supplementary Figure S2. Bioinformatic analysis workflow for IMP2-target detection from HyperTRIBE.

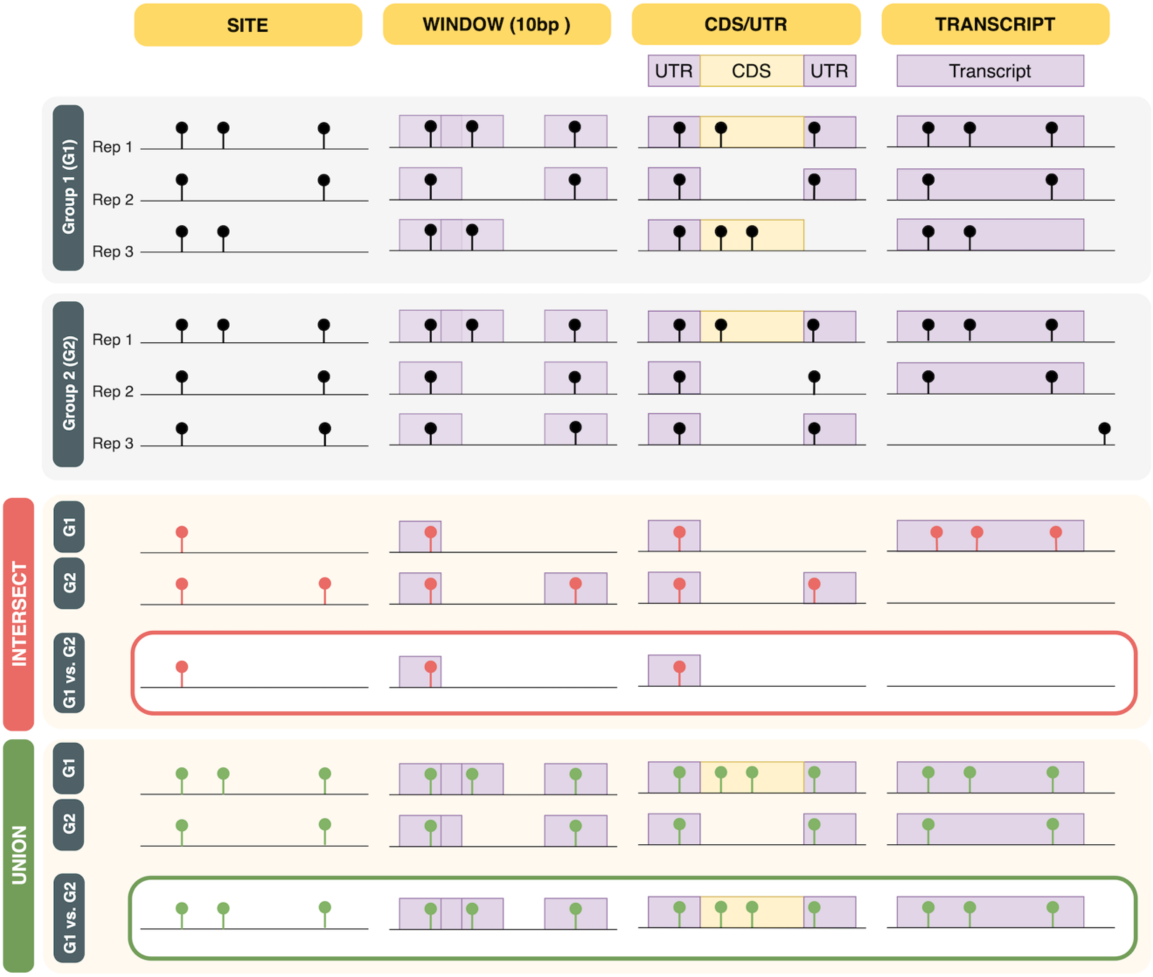

Supplementary Figure S3. Replicate collapsing schemes. Editing sites or regions are combined across replicates by different strategies. (Middle) The red-colored INTERSECT scheme requires a site/region to be present in all replicates of a sample to be included, whereas (bottom) the green-colored UNION only requires the in at least two replicates. Three different genomic regions for combining the editing sites were investigated, including an overlapping window of 10 bps, CDS or UTRs, or full transcripts. If a region contains at least one editing site in any replicate within a sample group, it will be retained or omitted according to the INTERSECT/UNION schemes.

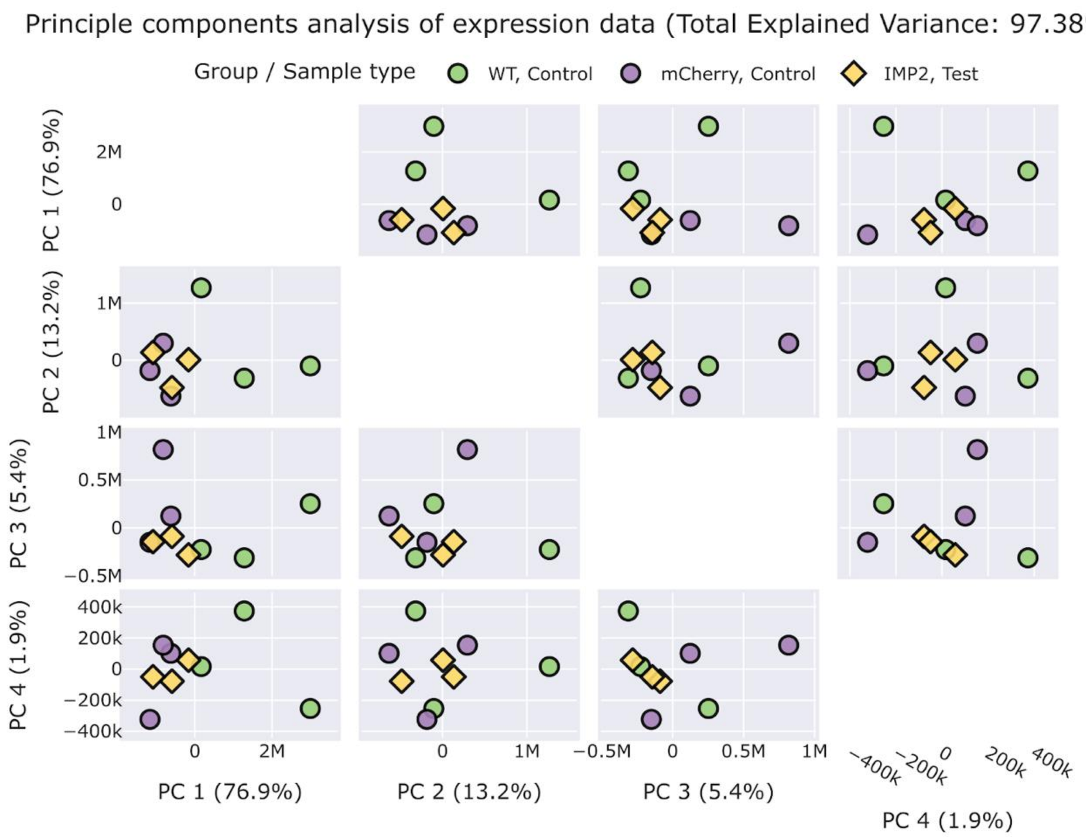

Supplementary Figure S4. Principal Component Analysis (PCA) of gene expression across samples. PCA was performed on the matrix containing raw gene expression levels from individual replicates of three sample groups, i.e., wild type (WT), mCherry, and IMP2. These expression data were transformed and clustered using the three (A) or four (B) largest principal components. The replicates are grouped by colors, while control samples, including WT and mCherry, and experiment samples (IMP2) are distinguished by round and diamond shapes, respectively.

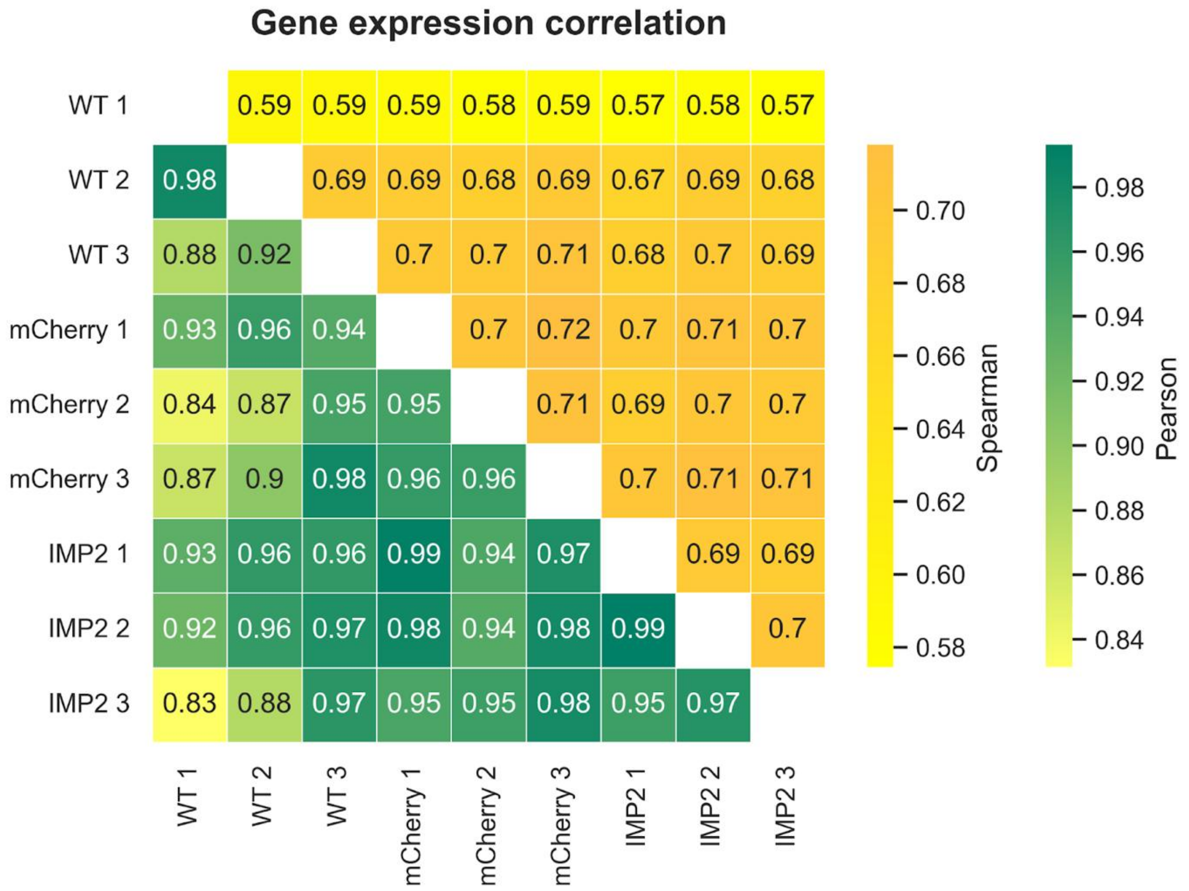

Supplementary Figure S5. Correlation analysis of gene expression across samples. The raw expression levels from different replicates were correlated using either Pearson (lower triangle) or Spearman (upper triangle) correlation. Correlation coefficients are represented by colors in the heatmap.

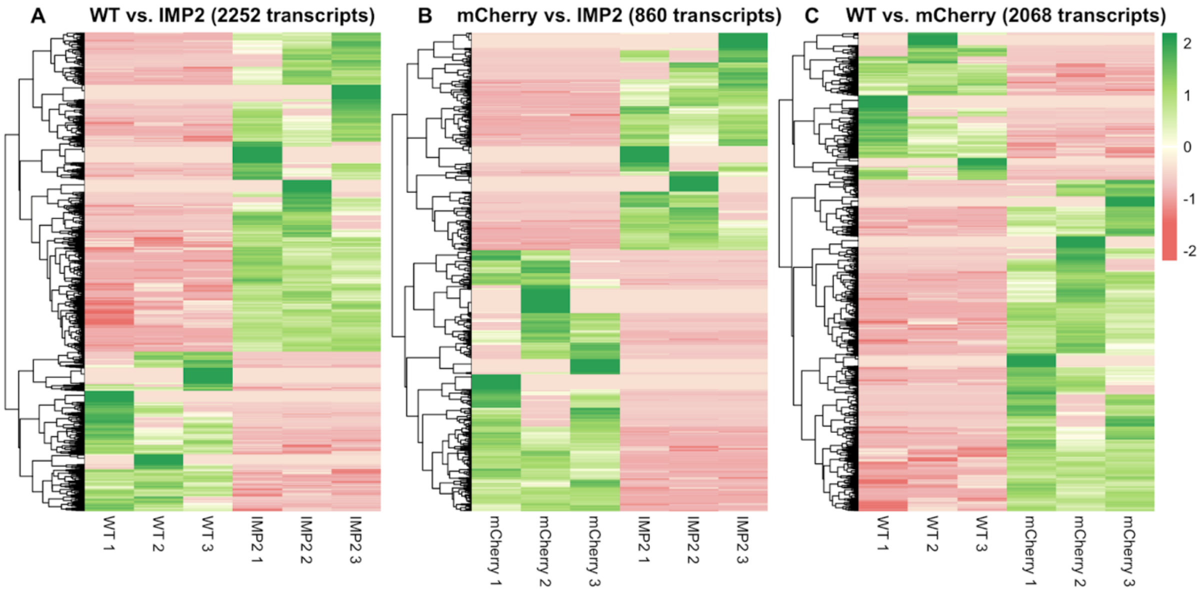

Supplementary Figure S6. Heatmaps for expression levels of differentially expressed genes (DEGs). Using DESeq2, the lists of DEGs specific for WT and IMP2 (A), mCherry and IMP2 (B), and WT and mCherry (C) were determined (|LFC| < 1, FDR-adjusted p-value < 0.05). Hierarchical clustering of DEGs shows clusters of up/down-regulated genes in the samples for each pairwise comparison.

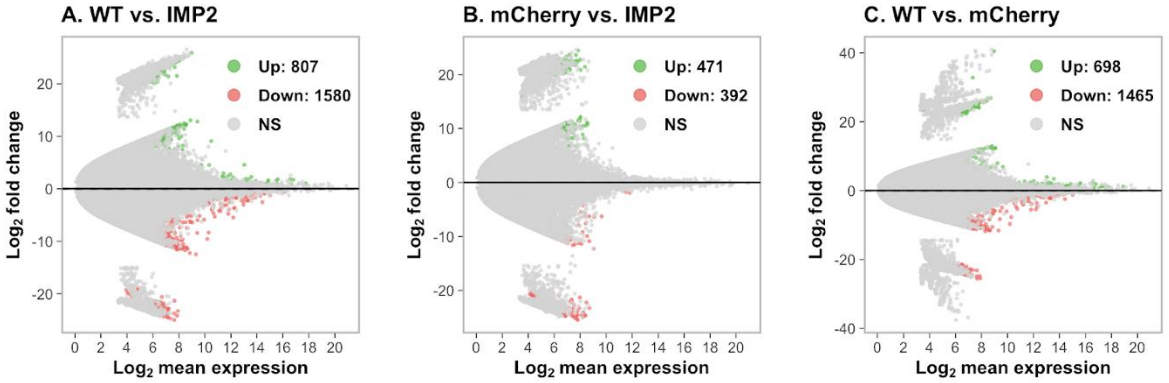

Supplementary Figure S7. MA plots for normalized count data from differential analysis. The gene expression data for each sample was normalized, log-transformed, and compared using DESeq2 in a pairwise manner. The log fold changes (LFCs) of individual genes are plotted against the mean of normalized gene expression in the comparisons between WT and IMP2 (A), mCherry and IMP2 (B), and WT and mCherry (C) samples. Genes with differential expression (|LFC| < 1, FDR-adjusted p-value < 0.05) between two sample groups are highlighted in green (up-regulated) or red (down-regulated), while other genes are depicted in gray color (NS).

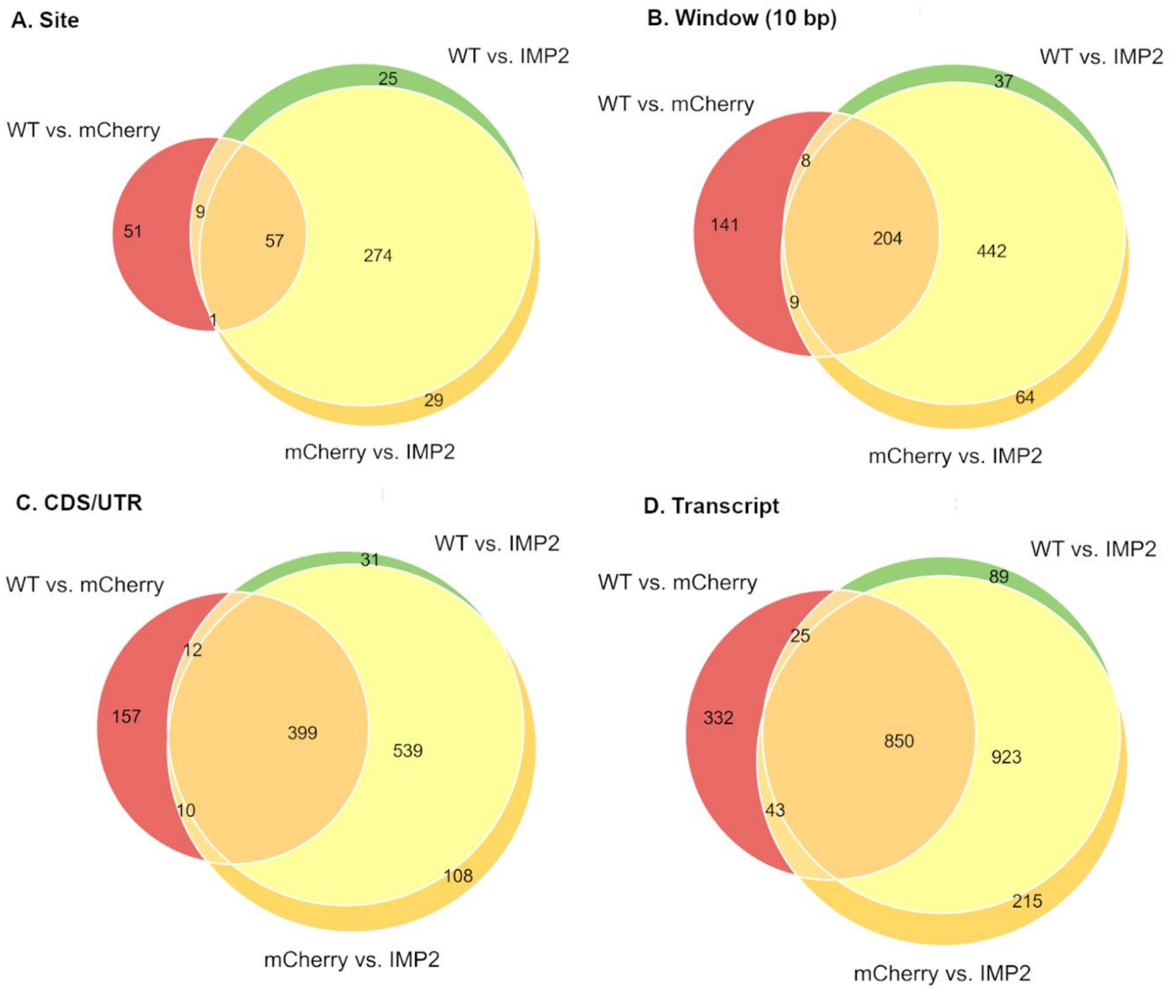

Supplementary Figure S8. Overlaps between the sets of genes containing A2G sites from HyperTRIBE pairwise comparisons. A2G sites were detected at 1% average editing percentage. A gene must contain at least one A2G site present in a specific genome region of at least two out of three replicates to be considered in the result set. In all four investigated regions, comparing the control groups (WT or mCherry) to the IMP2 group resulted in highly similar sets of IMP2 genes (A-D), whereas the IMP2 genes from comparing the control groups take up at least 16.96% from the union of these sets (A).

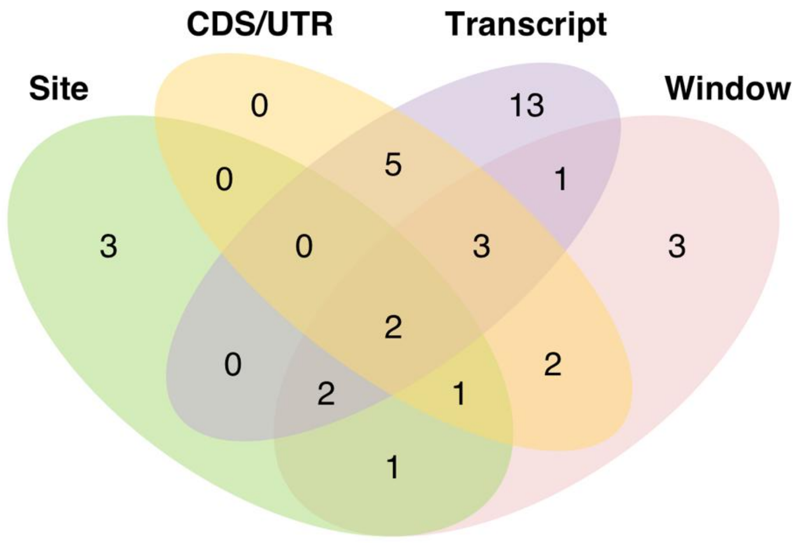

Supplementary Figure S9. Venn diagram of overlapping IMP2 genes based on A2G sites from four different genomic regions. IMP2 genes are genes with A2G sites which were identified from the comparison between any control group (WT or mCherry) and IMP2 group, but not in the comparison between WT and mCherry (Supplementary Table S5, column E). The A2G sites from all replicates within a sample group were gathered if they belong to the same genomic region (Supplementary Figure S3).

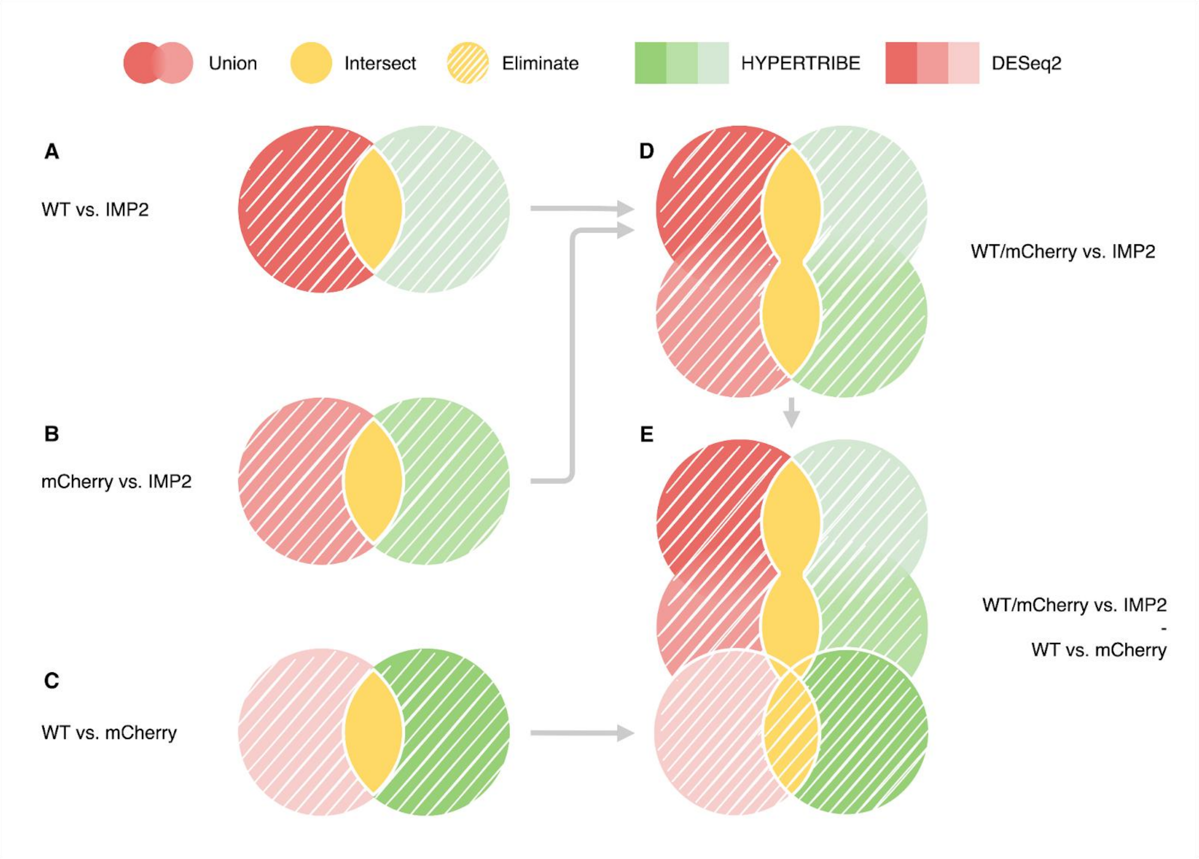

Supplementary Figure S10. Schematic for finding the overlap between HyperTRIBE and DESeq2 gene sets. The genes with both A2G sites and deregulated transcripts are collected for the comparisons between WT or mCherry and IMP2, or between WT and mCherry (A-C) and correspond to columns A-C in Supplementary Table S5. The unions of genes from *WT vs. IMP2* and *mCherry vs. IMP2* for HyperTRIBE and DESeq2 were overlapped and resulted in *WT/mCherry vs. IMP2* gene set (D - corresponding to Supplementary Table S5, column D). From the unions between *WT/mCherry vs. IMP2*, “control genes” found in *WT vs. mCherry* were eliminated, leaving the sets of “IMP2-related genes” for both HyperTRIBE and DESeq2 analysis. The intersection between those IMP2-related gene sets defines *WT/mCherry vs. IMP2 - WT vs. mCherry* group (E - corresponding to Supplementary Table S5, column E).

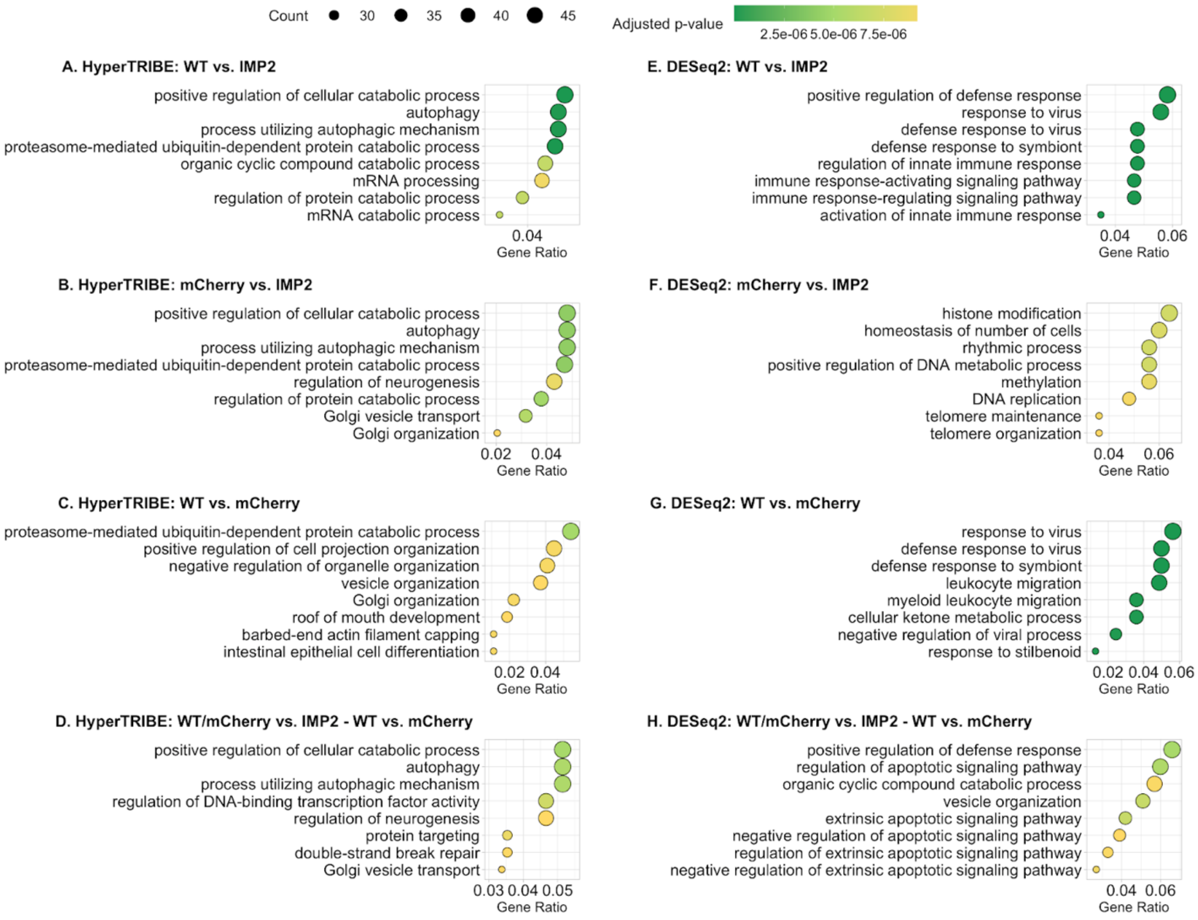

Supplementary Figure S11. Gene ontology enrichment analysis of IMP2 target genes (A-D) and differentially expressed genes (DEGs) (E-H). Shown are the most significantly enriched biological process terms (FDR ≤ 0.05) for comparisons between WT and IMP2 (A, E), mCherry and IMP2 (C, G), and for any comparison to IMP2, but not for the comparison between controls (D, H). Enrichments were computed with the R package *clusterProfiler.*

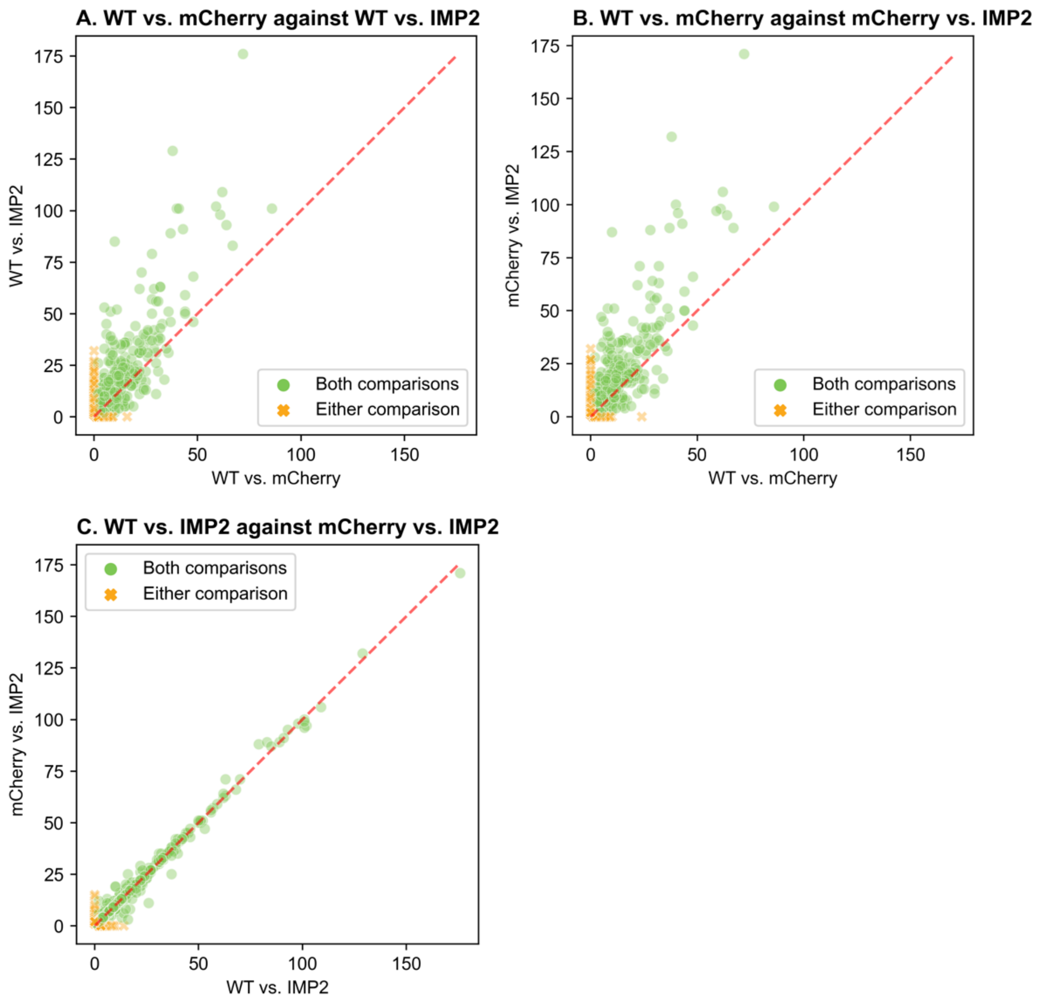

Supplementary Figure S12. Distribution of the number of A2G sites per gene (T=1%, UNION, CDS/UTR). For different pairs of comparisons, the genes with A2G sites are plotted using the number editing sites identified in each comparison. Genes containing editing sites only in a comparison are represented in yellow, while those with editing sites in both comparisons are depicted in green.

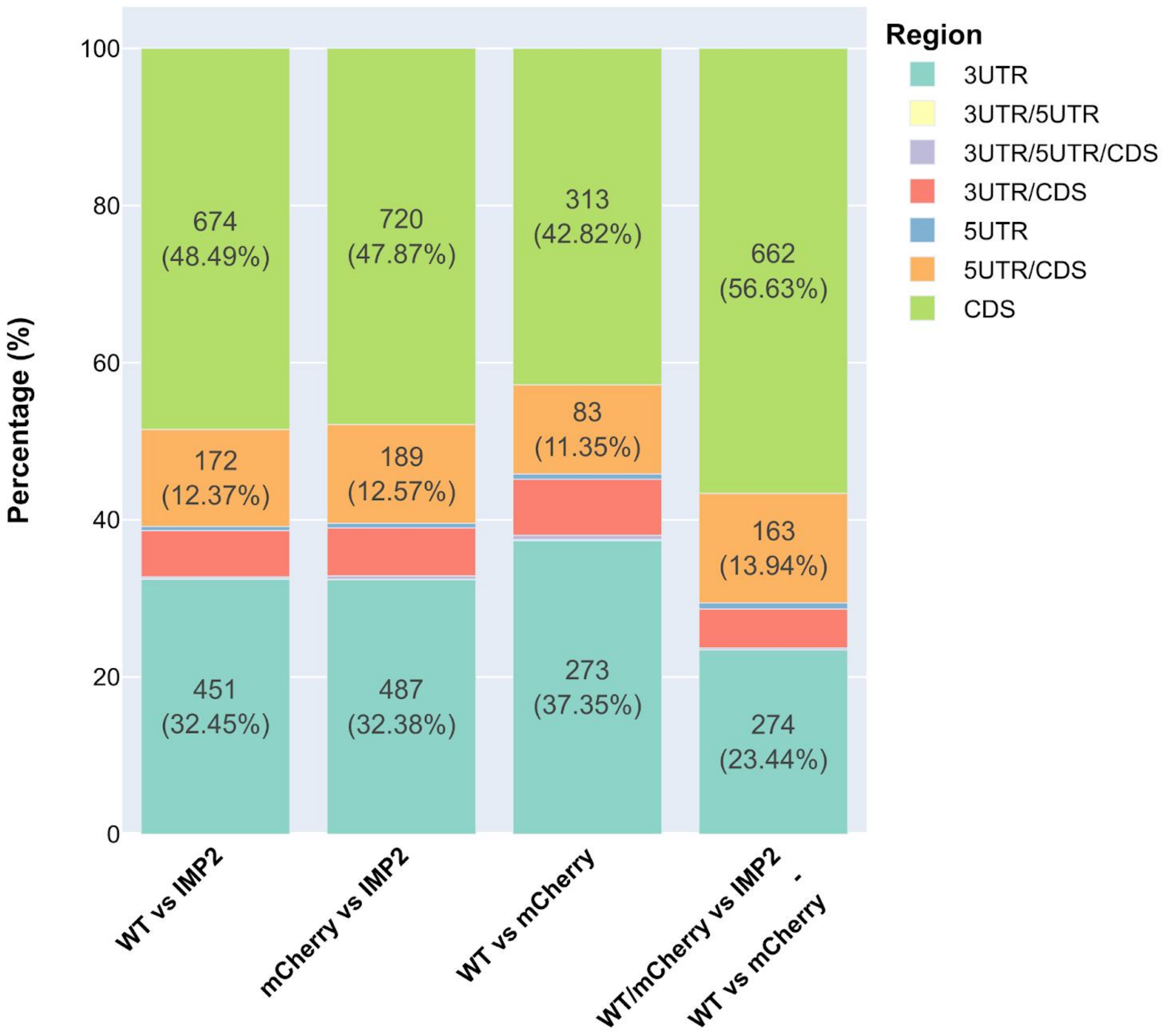

Supplementary Figure S13. Distribution of A2G sites remained for the selected replicate collapsing scheme (T=1%, UNION, CDS/UTR). Using the *Mus musculus* reference genome mm39, regions containing A2G sites could be annotated as CDS, 3'UTR or 5'UTR in a non-exclusive manner. The A2G sites detected at 1% average editing percentage and present in CDS or UTR regions of at least two out of three replicates were considered for the comparisons between IMP2 and either WT or mCherry (*WT vs. IMP2* and *mCherry vs. IMP2*), or between WT and mCherry (*WT vs. mCherry*). The *WT/mCherry vs. - WT vs. mCherry* group consists of CDSs or UTRs from either *WT vs. IMP2* or *mCherry vs. IMP2* that do not share any overlap with those in *WT vs. mCherry*.

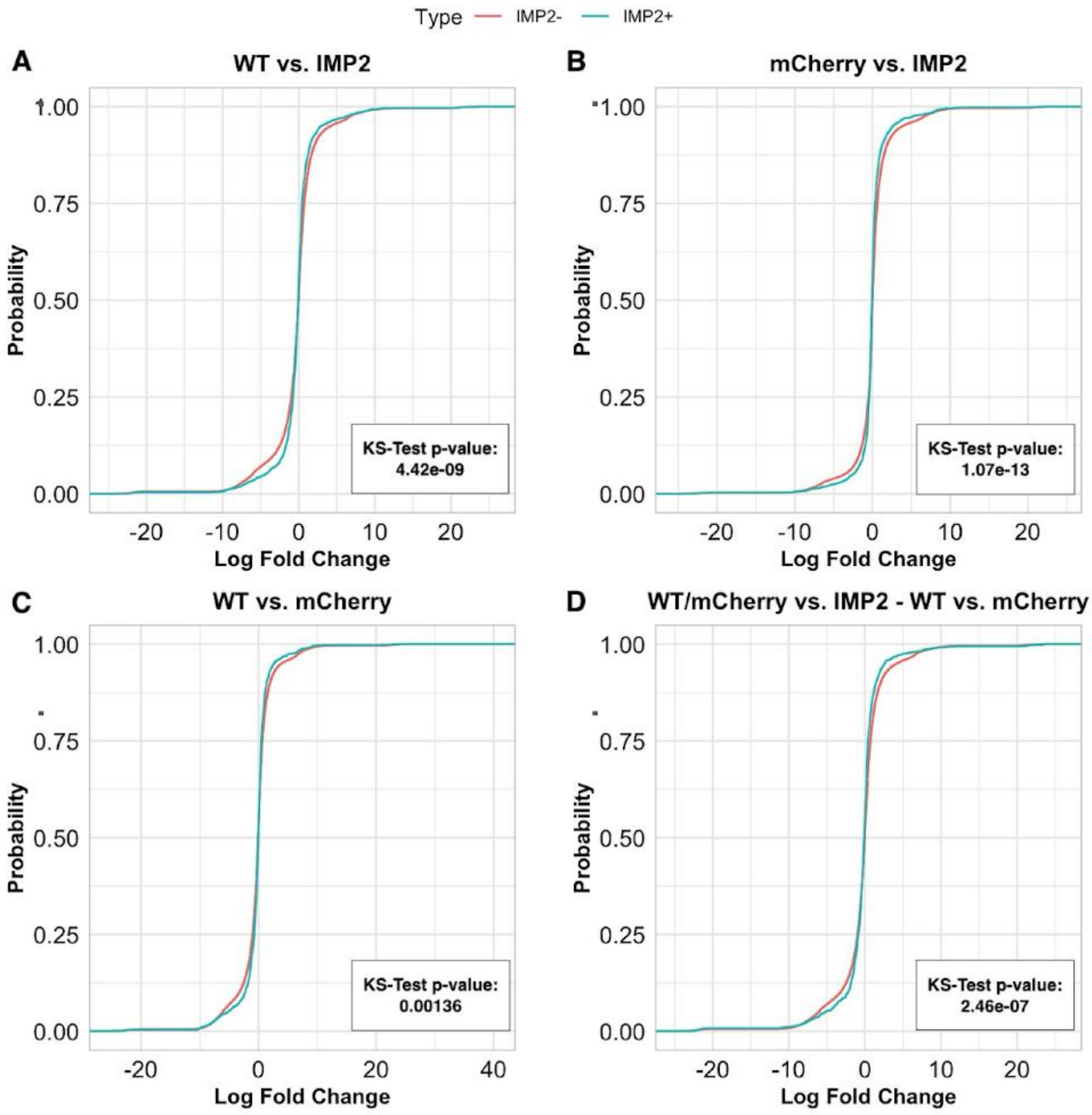

Supplementary Figure S14. Expression profiles and motif analysis of identified IMP2 target genes. (A-D) Cumulative distribution of Log Fold Change (LFC) for IMP2 target genes (T=1%, UNION, CDS/UTR). The cumulative LFC distributions for all genes computed by DESeq2 were plotted separately for genes with and without A2G sites (denoted by IMP2+ and IMP2- with blue and red colors, respectively) that were identified by HyperTRIBE. These LFCs quantify the change in gene expression levels between IMP2 and either WT or mCherry (A and B), or between WT and mCherry (C). (D) shows the IMP2+ set of genes with A2G sites detected in any comparison against IMP2 samples, excluding those belonging to the comparison between controls. Kolmogorov-Smirnow tests were used to compare the cumulative distribution between any IMP2- and IMP2+ set of LFCs.

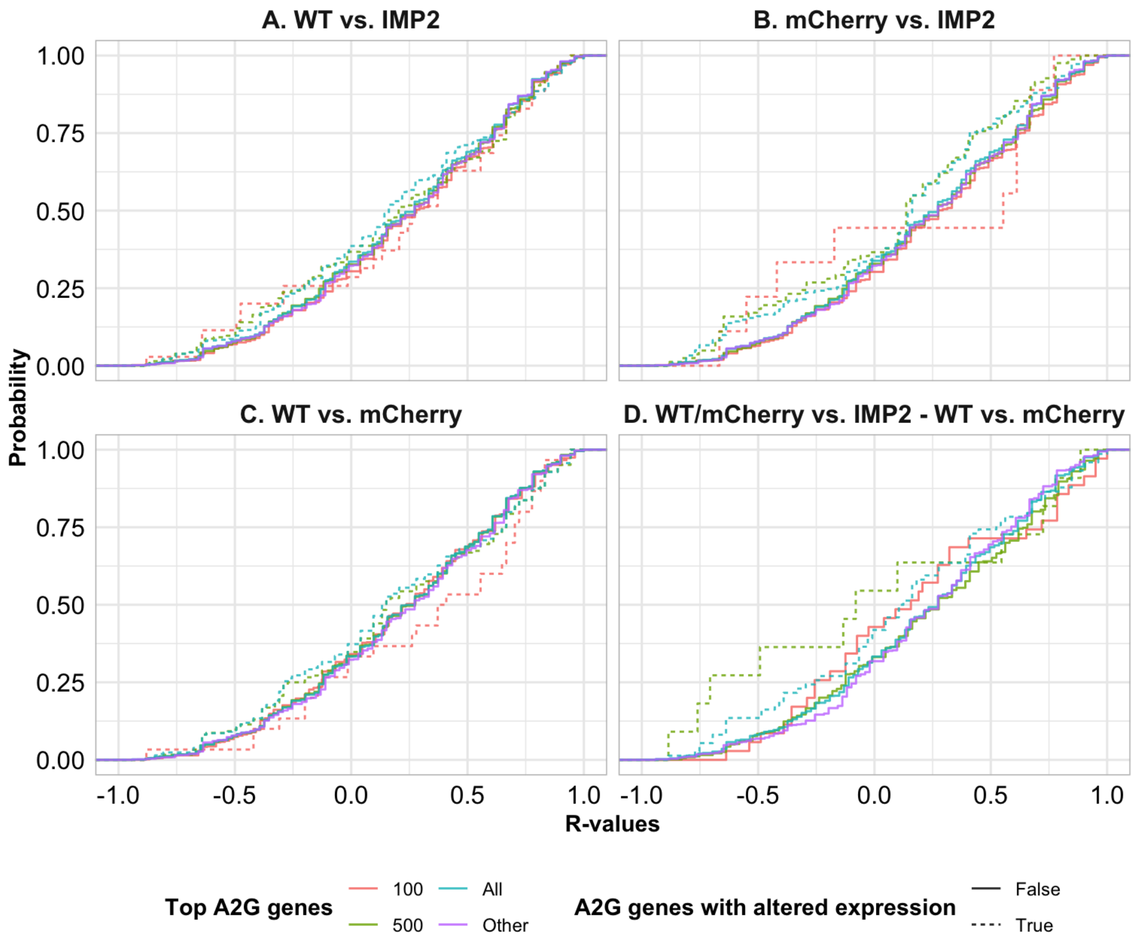

Supplementary Figure S15. Distribution of correlation coefficients in expression level between IMP2 and other genes. Spearman correlation between the expression levels of IMP2 and all other genes was computed and the empirical cumulative distribution of the correlation coefficients was plotted. The genes were categorized into and colored according to four groups, namely all genes with A2G sites (All), top 100 and 500 A2G genes with the highest editing percentage (100 and 500), and genes with no A2G sites (Other). The genes with A2G sites detected by HyperTRIBE are specific for each of the three comparisons, between WT or mCherry and IMP2 or between WT and mCherry. Genes in "*WT/mCherry vs. IMP2*" groups were detected in the WT or mCherry against IMP2 comparison, but not in the WT against mCherry comparison. The distributions for A2G genes with deregulated expression identified by DESeq2 listed in Supplementary Table S6 are plotted in dashed lines.

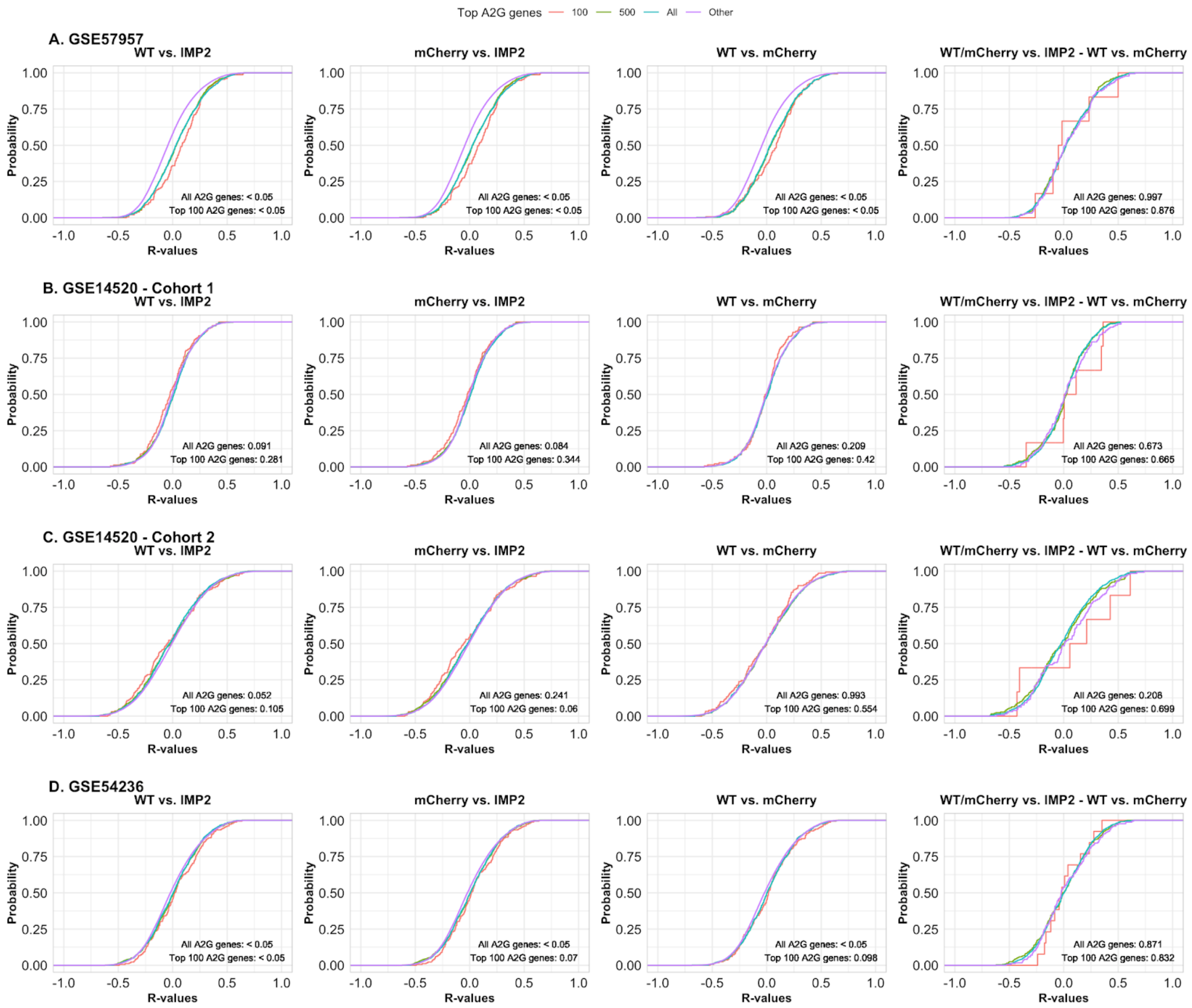

Supplementary Figure S16. Distribution of correlation coefficients in expression level between IMP2 and other genes in public datasets. We retrieved from Gene Expression Omnibus four datasets containing RNA-seq data for normal liver tissues in human patients (GSE57957, GSE14520 cohort 1 and 2, and GSE54236 with 59, 22, 42, and 76 samples, respectively). For each dataset, the Spearman correlation between the expression levels of IMP2 and all other genes was computed and the empirical cumulative distribution of the correlation coefficients was plotted. The genes were categorized into and colored according to four groups, namely all genes with A2G sites (All), top 100 and 500 A2G genes with the highest editing percentage (100 and 500), and genes with no A2G sites (Other). The genes with A2G sites detected by HyperTRIBE are specific for each of the three comparisons, between WT or mCherry and IMP2 or between WT and mCherry. Genes in "*WT/mCherry vs. IMP2*" groups were detected in the WT or mCherry against IMP2 comparison, but not in the WT against mCherry comparison. The Kolmogorow-Smirnow test was performed to compare the distribution between the top 100 A2G genes (100) or all A2G genes (All) to non-A2G genes (Other) and the p-values are reported in the bottom right corner of each panel.

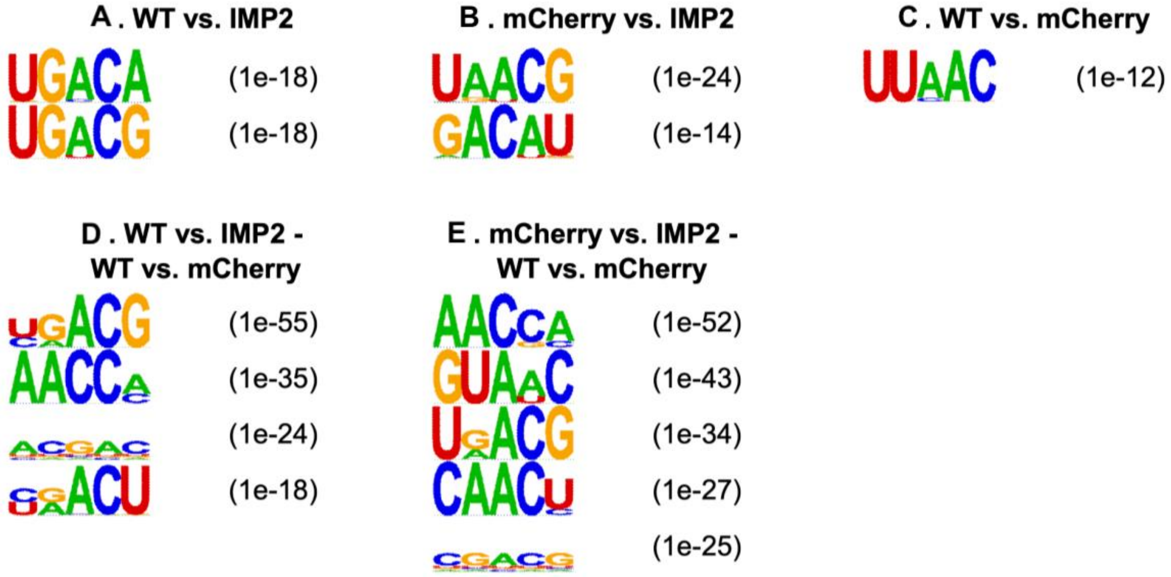

Supplementary Figure S17. m6A-related motifs enriched in IMP2 binding regions were identified using the HOMER Motif Discovery tool. *De novo* motif analysis was performed for sequences in a [-500 bp, +500 bp] region around each A2G site of each comparison group (*WT vs. IMP2*, *mCherry vs. IMP2* and *WT vs. mCherry* in Subfigure A, B, and C, respectively). In differential motif analysis, all positions identified in the *WT vs. mCherry* comparison were removed from the *WT vs. IMP2* (D) and *mCherry vs. IMP2* (E) comparisons. Significantly enriched motifs possess adjusted p-values smaller than 1e-10 and are tabulated in Supplementary File 3. Panels A to E show only motifs similar to RAC/RACH/DRACG variants of the m6A consensus motifs and their adjusted p-values (where D = A/G/U, R = A/G, and H = U/A/C).

Supplementary Tables

A	Gene name	NCBI accession number	Forward primer	Reverse primer	Annealing temp. (°C)	Primer conc. (µM)
	Igf2bp2 (murine)	NM_183029.2	TTGGATGGGCTGTTGGCTGA	GTGACGTTGACAACGGCAGTT	60	0.2
	Rn18s	NM_008907.1	AGGTCTGTGATGCCCTTAGA	GAATGGGGTTCAACGGGTTA	61	0.25
	IGF2BP2 (human)	NM_006548.6	GTTCCCGCATCATCACTCTTAT	GAATCTCGCCAGCTGTTTGA	62	0.2

B	Gene name	Forward primer	Reverse primer
	CREB1	GACCACTGATGGACAGCAGATC	GAGGATGCCATAACAACTCCAGG
	MED16	TCTCAACACGCCTGACAAGAGC	GTCCAGCACAAATTCCTCCGTC
	RABEP1	AGCAGCGAAGATTCGAGTCACC	CTGAACATCCCTCTTTGCCTGG
	TAF6	CCTGATGCGTATGGTGAAAGCG	ACATCTGGTCGCAGGCACAACT

Supplementary Table S1. Primer sequences for qPCR (A) and RNA immunoprecipitation (B).

	All A2G sites			1%			5%		
	WT vs. IMP2	mCherry vs. IMP2	WT vs. mCherry	WT vs. IMP2	mCherry vs. IMP2	WT vs. mCherry	WT vs. IMP2	mCherry vs. IMP2	WT vs. mCherry
**Total no. editing sites**	46,963	47,624	39,289	10,004	10,327	5,373	1,064	1,074	117
**Total no. transcripts**	2,294	2,413	1,803	981	1,056	578	173	186	23
**Average no. editing sites per transcript**	2.8	3.0	1.9	3.7	3.7	2.8	12.7	12.3	14.0

Supplementary Table S2. Total number of transcripts and editing sites detected by HyperTRIBE. We used HyperTRIBE to detect transcripts with editing sites by comparing IMP2 to either one of the controls (WT or mCherry) or comparing the controls against each other. The identified editing sites with read coverage higher than 20 reads and passing an average editing threshold (1% or 5% in all transcripts) are listed in Supplementary File 1. Here, we summarized the total number of editing sites identified for each comparison and each editing threshold, together with the total number of transcripts containing these editing sites and the average editing percentage per transcript. The columns in “All A2G sites” show the number of sites, transcripts and average editing sites per transcript when no editing threshold filter is applied.

	INTERSECT						UNION					
	1%			5%			1%			5%		
	WT vs. IMP2	mCherry vs. IMP2	WT vs. mCherry	WT vs. IMP2	mCherry vs. IMP2	WT vs. mCherry	WT vs. IMP2	mCherry vs. IMP2	WT vs. mCherry	WT vs. IMP2	mCherry vs. IMP2	WT vs. mCherry
**Editing Site**	276	321	96	59	55	8	366	362	119	83	77	14d
**Overlapping gene span (10bp)**	500	635	315	68	66	10	692	720	363	95	93	14
**CDS/UTR**	715	976	532	111	167	18	982	1,057	579	174	187	24
**Transcript**	1,347	1,837	1,148	170	259	29	1,888	2,032	1,251	283	303	44

Supplementary Table S3. Number of genes with A2G sites for different replicate collapsing schemes. Using HyperTRIBE, we identified and compared the editing sites between IMP2 and either one of the controls (WT or mCherry) or between the controls. Listed here are gene counts with either exactly overlapping editing sites or showing overlap in a certain region (10 bp window, CDS or UTR regions, and transcript) that were identified either in all replicates of a sample group (INTERSECT) or in at least two out of three replicates (UNION). Two different thresholds were applied for the average editing percentage (1% and 5%). Overall, we identified more genes containing at least one A2G site when we considered less stringent thresholds for average editing percentage (1%). As TRIBE is not able to reveal the exact binding position of the RBP on the mRNA (28), retaining only the editing sites identified in all three replicates of one condition (INTERSECT) can therefore be considered as a fairly strict filter, which may cause loss of information. Finally, we selected the genomic span for the final replicate collapsing scheme by comparing the sets of IMP2 targets identified in this analysis to those in mouse embryonic fibroblasts, or the set of differentially expressed genes detected by DESeq2.

	INTERSECT						UNION					
	1%			5%			1%			5%		
	WT - IMP2	mCherry - IMP2	WT - mCherry	WT - IMP2	mCherry - IMP2	WT - mCherry	WT - IMP2	mCherry - IMP2	WT - mCherry	WT - IMP2	mCherry - IMP2	WT - mCherry
**Editing Site**	173 (62.7 %)	195 (60.8 %)	67 (69.8 %)	33 (56.0 %)	26 (47.3 %)	1 (12.5 %)	219 (59.8 %)	215 (59.4 %)	80 (67.2 %)	36 (43.4 %)	32 (41.6 %)	5 (35.7 %)
**Overlapping gene span (10bp)**	320 (64.0 %)	388 (61.1 %)	205 (65.1 %)	36 (54.6 %)	34 (50.0 %)	2 (20.0%)	424 (61.3 %)	435 (60.4 %)	233 (64.2 %)	41 (43.2 %)	41 (44.1 %)	5 (35.7 %)
**CDS/UTR**	443 (62.0 %)	594 (60.9 %)	363 (68.2 %)	59 (53.2 %)	87 (52.1 %)	7 (38.9 %)	595 (60.6 %)	634 (60.0 %)	386 (66.7%)	84 (48.3 %)	94 (50.3 %)	12 (50.0 %)
**Transcript**	756 (56.1 %)	1,019 (55.5 %)	692 (60.3 %)	7 (38.9 %)	116 (44.8 %)	10 (34.5 %)	1,048 (55.5 %)	1,118 (55.0 %)	739 (59.1 %)	121 (42.8 %)	131 (43.2 %)	15 (34.1 %)

Supplementary Table S4. The number and percentage of genes with A2G sites overlapping with HyperTRIBE results from mouse embryonic fibroblast (MEF) samples. For each replicate collapsing scheme, the set of genes with at least one editing site was compared to the HyperTRIBE results of MEF samples, which used the same average editing percentage thresholds of 1% and 5%. The number of overlapping genes between the two sets, as well as their percentage in all genes found with A2G sites, are reported for the comparisons between IMP2 and either controls (WT or mCherry) or between the controls. The overlap was quite small when using a 5% threshold for the average editing percentage. Thus, we selected the results from the 1% threshold to ensure better interpretability and robustness.

	WT *vs.* IMP2	mCherry *vs.* IMP2	WT *vs.* mCherry	WT/mCherry *vs.* IMP2	WT/mCherry *vs.* IMP2 - WT *vs.* mCherry (IMP2 genes)	WT/mCherry *vs.* IMP2 - WT *vs.* mCherry / WT/mCherry *vs.* IMP2
	**A = HTRIBE ∩ DEG**	**B = HTRIBE ∩ DEG**	**C = HTRIBE ∩ DEG**	**D = HTRIBE(WT *vs.* IMP2 ∪ mCherry *vs.* IMP2) ∩ DEG (WT *vs.* IMP2 ∪ mCherry *vs.* IMP2)**	**E = HTRIBE(WT *vs.* IMP2 ∪ mCherry vs. IMP2 - WT *vs.* mCherry) ∩ DEG (WT *vs.* IMP2 ∪ mCherry *vs.* IMP2 - WT *vs.* mCherry)**	**F = E/D**
**Site**	13.40 (17)	11.31 (7)	4.23 (4)	14.86 (21)	13.16 (9)	0.43
**Window**	20.25 (32)	12.35 (12)	13.58 (16)	26.18 (46)	16.8 (15)	0.33
**CDS/UTR**	27.55 (51)	12.26 (16)	17.32 (24)	32.85 (68)	12.62 (13)	0.19
**Transcript**	39.61 (107)	15.03 (34)	25.62 (52)	45.31 (138)	16.6 (26)	0.19

Supplementary Table S5. Similarity between genes with A2G sites and differentially expressed genes (T=1%, UNION). The overlap between genes with A2G sites from HyperTRIBE analysis and genes with differential expression from DESeq2 analysis was measured using Jaccard Index x 1000 (columns A-E) and is reported as overlapping gene count. Both analyses compared IMP2 to either controls (WT or mCherry in columns A and B), or WT to mCherry (C). Similarly, we computed the overlap between HyperTRIBE and DESeq2 results, specifically for the union sets of *WT vs. IMP2* and *mCherry vs. IMP*2 results (column D) and without genes in *WT vs. mCherry* result (column E). The last column (F) lists the fractions of genes showing up only in the comparisons with IMP2 samples and not in *WT vs. mCherry*. Supplementary Figure S10 illustrates how gene sets were joined. Here, we considered A2G sites/regions detected at 1% average editing percentage and that are present in at least two out of three replicates. The respective gene names are listed in Supplementary Table S6. The results for CDS/UTR in this table and the corresponding gene names in Supplementary Table S6 are presented in Table 1 in the manuscript. Supplementary Figure S9 shows the overlaps between IMP2-gene sets from different genomic regions.

**Comparison**	**Genes**
**WT *vs*. IMP2**	Calu, Ccdc90b, Cdadc1, Clta, Cped1, Cplane1, Creb1, Crem, Cyp2c38, Dck, Ddx11, Ddx58, Dhx58, Dlg1, Ecm1, Elmod3, Esam, Fktn, Gm20604, Grk6, Hck, Igtp, Macf1, Mad2l2, Me2, Met, Mff, Mmrn2, Mtif3, Mug1, Numb, Parp14, Pecam1, Plec, Ppil3, Rabep1, Reln, Retreg1, Rnf38, Slc66a2, Stx5a, Syt12, Taf6, Tardbp, Tdrd7, Tgtp1, Tmem62, Tnpo1, Trim39, Vps39, Wnk1
**mCherry *vs*. IMP2**	Creb1, Crem, Fam219a, Fktn, Gm20604, Map7d1, Med16, Nfix, Prpf40b, Ptbp3, Rabep1, Smim14, Taf6, Tardbp, Tnpo1, Vps39
**WT *vs*. mCherry**	Add3, Cdadc1, Cyp27a1, Dhx58, Dlg1, Dusp12, Fktn, Gm11837, Hsd3b5, Lrp11, Mmrn2, Oasl1, Pigw, Plec, Pnpt1, Prpf40b, Retreg1, Rnf38, Slfn4, Tdrd7, Ttbk2, Ubr5, Wnk1, Zfp708
**WT/mCherry *vs*. IMP2 - WT *vs*. mCherry**	Agfg1, Ccdc90b, Creb1, Cyp2c38, Dck, Mad2l2, Med16, Rabep1, Taf6, Tardbp, Tmem62, Tnpo1, Trim39

Supplementary Table S6. Genes with deregulated transcript levels and identified as IMP2 targets. Genes with A2G sites from HyperTRIBE analysis (using 1% average editing threshold, combined for mutual CDS/UTR across at least two replicates) and differential expression from DESeq2 analysis (|LFC| < 1, FDR-adjusted p-value < 0.05) are summarized for the comparison between IMP2 and either controls (WT or mCherry) or WT and mCherry. The genes in the last row are genes identified in the comparison between any control and IMP2, but not in the *WT vs. mCherry* comparison.

	WT vs. IMP2	mCherry vs. IMP2	WT vs. mCherry
**WT vs. IMP2**	-	-	-
**mCherry vs. IMP2**	0.14	-	-
**WT vs. mCherry**	0.49	0.13	-

Supplementary Table S7. Similarity between differentially expressed genes. Differential expression analysis between each pair of samples was performed with DESeq2. The sets of deregulated genes from the pairwise comparisons were compared using Jaccard indices.
